# Biochemical and Immunological Characterization of Truncated Fragments of the Receptor-Binding Domains of *C. difficile* Toxin A

**DOI:** 10.1371/journal.pone.0135045

**Published:** 2015-08-13

**Authors:** Jui-Hsin Huang, Zhe-Qing Shen, Shu-Pei Lien, Kuang-Nan Hsiao, Chih-Hsiang Leng, Chi-Chang Chen, Leung-Kei Siu, Pele Choi-Sing Chong

**Affiliations:** 1 Vaccine R&D Center, National Institute of Infectious Diseases and Vaccinology, National Health Research Institutes, Zhunan Town, Miaoli County, Taiwan; 2 Graduate Institute of Life Science, National Defense Medical Center, Taipei, Taiwan; 3 Graduate Institute of Immunology, China Medical University, Taichung, Taiwan; University of Arizona, UNITED STATES

## Abstract

*Clostridium difficile* is an emerging pathogen responsible for opportunistic infections in hospitals worldwide and is the main cause of antibiotic-associated pseudo-membranous colitis and diarrhea in humans. Clostridial toxins A and B (TcdA and TcdB) specifically bind to unknown glycoprotein(s) on the surface of epithelial cells in the host intestine, disrupting the intestinal barrier and ultimately leading to acute inflammation and diarrhea. The C-terminal receptor-binding domain (RBD) of TcdA, which is responsible for the initial binding of the toxin to host glycoproteins, has been predicted to contain 7 potential oligosaccharide-binding sites. To study the specific roles and functions of these 7 putative lectin-like binding regions, a consensus sequence of TcdA RBD derived from different *C*. *difficile* strains deposited in the NCBI protein database and three truncated fragments corresponding to the N-terminal (residues 1–411), middle (residues 296–701), and C-terminal portions (residues 524–911) of the RBD (F1, F2 and F3, respectively) were designed and expressed in *Escherichia coli*. In this study, the recombinant RBD (rRBD) and its truncated fragments were purified, characterized biologically and found to have the following similar properties: (a) are capable of binding to the cell surface of both Vero and Caco-2 cells; (b) possess Toll-like receptor agonist-like adjuvant activities that can activate dendritic cell maturation and increase the secretion of pro-inflammatory cytokines; and (c) function as potent adjuvants in the intramuscular immunization route to enhance immune responses against weak immunogens. Although F1, F2 and F3 have similar repetitive amino acid sequences and putative oligosaccharide-binding domains, they do not possess the same biological and immunological properties: (i) TcdA rRBD and its fragments bind to the cell surface, but only TcdA rRBD and F3 internalize into Vero cells within 15 min; (ii) the fragments exhibit various levels of hemagglutinin (HA) activity, with the exception of the F1 fragment, which demonstrates no HA activity; and (iii) in the presence of alum, all fragments elicit various levels of anti-toxin A-neutralizing antibody responses, but those neutralizing antibodies elicited by F2 did not protect mice against a TcdA challenge. Because TcdA rRBD, F1 and F3 formulated with alum can elicit immune protective responses against the cytotoxicity of TcdA, they represent potential components of future candidate vaccines against *C*. *difficile*-associated diseases.

## Introduction

Opportunistic nosocomial infection in hospitalized patients is often related to *Clostridium difficile* infection (CDI) that develops via disruption of the balance of the intestinal micro-flora by antibiotic therapies used during hospitalization. Thus, CDI often results in *Clostridium difficile*-associated diseases (CDAD), such as diarrhea, pseudomembranous colitis, and toxic megacolon [1,2]. In the past two decades, CDI has become a serious emerging infectious disease worldwide due to a significant increase in multi-drug resistance [3]. Moreover, the discovery of a hyper-virulent and antibiotic-resistant epidemic strain, NAP1/027, in developed countries poses a major challenge for CDAD prevention [4,5]. More importantly, *C*. *difficile* relapse is approximately 15–35% within a few weeks despite standard CDI therapy employing either vancomycin or metronidazole [6]. The pathogenicity of CDI is largely correlated to the clostridial toxins, toxin A and toxin B (TcdA and TcdB), that are secreted in the gastrointestinal environment of infected hosts and disrupt epithelial cell barriers in the small intestine [7]. Both toxins consist of a holotoxin with multi-functional domains that mediate *C*. *difficile* pathogenesis. The mechanism underlying TcdA and TcdB toxicity involves three steps: (a) binding to an unidentified receptor protein(s) on the surface of the intestinal epithelium and internalization through its C-terminal receptor-binding domain, (b) auto-cleavage and translocation of the N-terminal glucosyltransferase domain into the cytosol from the endosomal membrane; and (c) use of the N-terminal enzymatic region to inactivate the Rho GTPase family via glycosylation [8–10].

The published literature has indicated that TcdA-specific antibodies in patient sera positively correlated with the prevention of CDAD recurrence [11–15]. Therefore, passive immunization with anti-toxin antibodies has been shown to confer protection against CDI in murine models, and TcdA-specific monoclonal antibodies are currently being tested in clinical trials [11,16–19]. In addition, different *C*. *difficile* vaccine strategies are being evaluated; the most advanced strategy is vaccination with formalin-inactivated toxins [11, 20–21]. Immunization using the receptor-binding domain (RBD) of *C*. *difficile* toxins as the antigen in formulation with different adjuvants has been shown to elicit toxin-neutralizing antibody responses and protect mice against toxin or bacteria challenges [22–29]. The RBD is predicted to have a molecular size of approximately 100 kDa and is composed of 32–38 homologous repetitive peptides, depending on the sequence analysis [30–31]. Based on the crystal structure, the RBD was predicted to consist of 32 short repeat and 7 long repeat carbohydrate-binding sites [31]. The specific roles and functions of the 7 putative carbohydrate-binding regions are unclear, but they correlate to the binding of the oligosaccharide Galα1-3Galβ1-4GlcNAc [32–35]. Greco et al. [32] was the first to localize carbohydrate binding to the junction of 2 short repeats and a long repeat. The TcdB RBD has approximately 530 amino acids and 4 putative oligosaccharide-binding sites [33]. Interestingly, among *C*. *difficile* strains deposited in the NCBI database, the amino acid sequences of the putative oligosaccharide-binding sites between TcdA and TcdB were found to share approximately 50 to 70% similarity [36]. To this end, we rationally designed two novel immunogens based on these putative oligosaccharide-binding sites of TcdA RBD and TcdB RBD to induce broadly neutralizing antibodies against both toxins. The biochemical and immunological functions of the TcdB RBD have been characterized and published [36]. In this study, a recombinant TcdA RBD (rRBD) containing a consensus sequence of TcdA RBD identified from different *C*. *difficile* strains deposited in the NCBI protein database and three fragments corresponding to the N-terminal, middle, and C-terminal parts of RBD (F1, F2 and F3, respectively) were designed and expressed in *Escherichia coli*. The purified TcdA rRBD and its fragments were characterized biologically to establish a platform to study the specific roles and functions of the 7 putative receptor-binding regions. Mouse immunogenicity studies were performed to investigate the potential of the rRBD and/or its fragments as components of candidate vaccines against CDAD.

## Materials and Methods

### Ethics Statement

All experiments were conducted in accordance with the guidelines of the Laboratory Animal Center of the National Health Research Institutes (NHRI). Animal use protocols were reviewed and approved by the Institutional Animal Care and Use Committee of the National Health Research Institutes (approved protocol no. NHRI-IACUC-100053-A).

### Design and construction of the consensus sequence of rRBD and its truncated fragments

**Fig 1:**
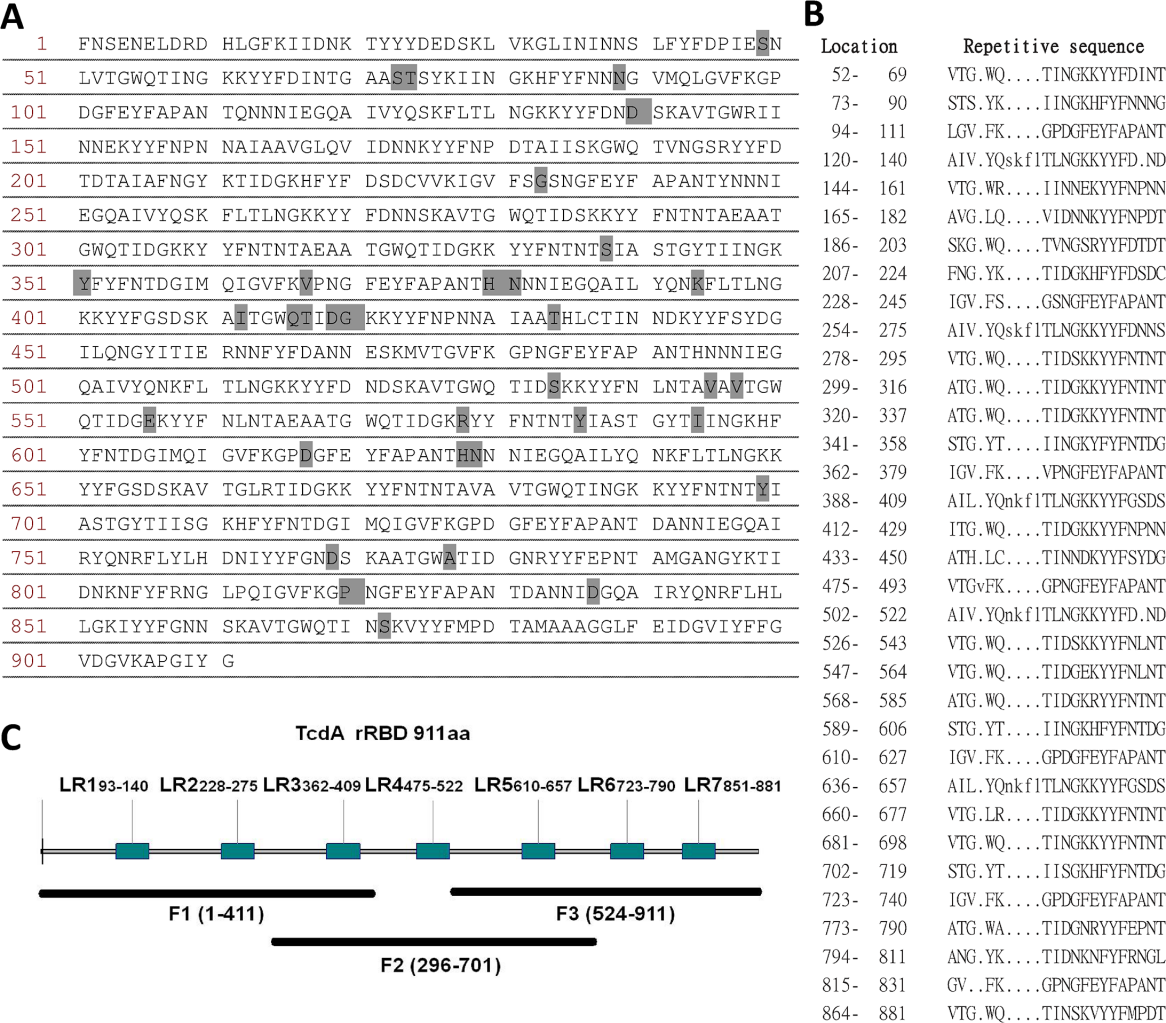
The consensus sequence of the C-terminal repeats and putative receptor-binding domain from *C*. *difficile* toxin A (TcdA rRBD). (A) The amino acid sequence (911 residues) of TcdA rRBD was identified using online software (http://www.ebi.ac.uk/Tools/pfa/radar/). Sequence alignment with a reference TcdA (strain VPI10463) was performed, and the sequence differences are highlighted. (B) The localization and sequence of each short repeat are represented in the left and right columns. (C) The RBD fragments are F1 (residues 1–411), F2 (residues 296–701) and F3 (residues 524–911); the 7 lectin-like receptor-binding (LR) sites are putatively located at residues 93–140 (LR1), 228–275 (LR2), 362–409 (LR3), 475–522 (LR4), 610–657 (LR5), 723–790 (LR6) and 815–881 (LR7).

RBD sequences from different *Clostridium difficile* strains deposited in the NCBI database were aligned for sequence analysis using the alignment tools from Vector NTI Advance 11.5 (Life Technologies, Carlsbad, CA). This consensus sequence was analyzed with online software (http://www.ebi.ac.uk/Tools/pfa/radar/) to detect repetitive protein sequences and predicted potential ligand-binding sites. The nucleotide sequence of TcdA rRBD was optimized for *E*. *coli* codon usage, chemically synthesized (GeneArt; Life Technologies) for cloning and expressed in *E*. *coli*. TcdA rRBD was inserted into a pET-22b vector (Novagen, Darmstadt, Germany) containing a poly-histidine tag at the 3’-end between NdeI and XhoI restriction sites. The resulting pET-22b_TcdA RBD construct was transformed into *E*. *coli* JM109 (DE3) (Promega, Madison, WI) for TcdA rRBD expression. TcdA rRBD was divided into three fragments, F1, F2 and F3, which correspond to the N-terminal residues 1 to 411, the middle region residues 296 to 701, and the C-terminal residues 524 to 911 of TcdA RBD (Fig 1A), respectively. The nucleotide sequences of the TcdA rRBD fragments containing a poly-histidine tag at the 3’-end were cloned into the pET-22b vector and transformed into *E*. *coli* JM109 (DE3) strain for protein expression.

### Purification of rRBD and its truncated fragments

TcdA rRBD and its truncated fragments were individually expressed in *E*. *coli* JM109 (DE3) (Promega) grown in Luria-Bertani medium (LB medium) at 20°C for 16 h following induction with 1 mM isopropyl-β-D-thiogalacto-pyranoside (IPTG). The purification process for TcdA rRBD and its fragments is briefly described below. Cells from 2 liters of culture medium were harvested by centrifugation and stored at -20°C before re-suspension in lysis buffer (50 mM sodium phosphate buffer, pH 7.2, containing 250 mM NaCl and 5 mM imidazole). The cells were physically disrupted using a French Press (Constant System, Daventry, UK) at 27 kpsi, and the supernatant was collected by filtration through a 0.22-μm filter. The crude extract was directly applied onto a nickel affinity chromatography column (GE Healthcare, Uppsala, Sweden) for purification of TcdA rRBD at 4°C. After sequential washes with a low concentration of imidazole buffer, TcdA rRBD was eluted using a lysis buffer containing 500 mM imidazole. The eluent was then dialyzed in a 30-kDa cut-off dialysis bag against phosphate buffered saline (PBS), pH 7.2, containing 10% glycerol. To remove bacterial endotoxin, the TcdA rRBD solution was passed through an E membrane (Pall Corporation, Ann Arbor, MI). All purification steps were analyzed by 8% SDS-PAGE. The residual endotoxin was determined using the Limulus amoebocyte lysate (LAL) assay (Associates of Cape Cod, Inc., Cape Cod, MA).

### Cell binding assay

Vero cells were seeded and cultured in 24-well plates using serum-free medium (VP-SFM) (Gibco, Carlsbad, CA) containing 4 mM glutamine at 37°C with 5% CO_2_, and the cells were grown to confluence. Varying amounts of TcdA rRBD were added to the wells, and PBS was used as the negative control. After a 30-min incubation, the wells were washed twice with cold PBS. Radioimmunoprecipitation assay buffer (RIPA buffer) (Millipore, Billerica, MA), a strong cell lysis buffer, was added to the cells. The total cell lysate was analyzed by SDS-PAGE and then transferred onto polyvinylidene difluoride (PVDF) membranes (Pall Corporation) pre-soaked in methanol. PVDF membranes were blocked with 5% (w/v) nonfat dry milk in PBS and sequentially exposed to 0.5 μg/mL TcdA-specific monoclonal antibodies (Clone PCG-4; GeneTex, Taiwan) and 50 ng/mL horseradish peroxidase (HRP) conjugated to a secondary antibody (GeneTex) to detect the amount of TcdA rRBD bound to Vero cells. Antibodies were diluted in PBS containing 1% nonfat dry milk (w/v). PVDF membranes were washed twice at every step with PBST (1× PBS containing 0.05% Tween 20) at room temperature. Finally, signals were detected using enhanced chemiluminescence (ECL) according to the manufacturer’s instructions (KPL, Gaithersburg, MD).

### Immunofluorescence staining

Vero cells seeded in T-75 flasks with VP-SFM containing 4 mM glutamine at 37°C and 5% CO_2_ were allowed to grow to 80% confluence. Cells were isolated and suspended with VP-SFM culture medium at 2 × 10^5^ cells/mL inside a flow tube. Various amounts of either TcdA rRBD or its fragments were mixed with the cells and incubated at 37°C for 5, 15, and 30 min, and cell binding was stopped at 4°C. The cells were fixed and then treated with specific buffers for flow analysis (eBioscience, San Diego, CA). After washing three times with cold PBST buffer, 1 μg/mL goat anti-Fc receptor antibody (BD Science) was added for 10 min to prevent any non-specific binding of antibodies. The cells were washed with cold PBST three times before stepwise incubations with 1 μg/mL anti-TcdA mouse monoclonal antibody PCG-4 (GeneTex) and 1 μg/mL secondary antibody (goat anti-Fc receptor antibody) conjugated to fluorescein isothiocyanate (FITC) (Sigma-Aldrich). After the last washing step, the cells were washed with double distilled H_2_O to remove the salt. The cells were simultaneously mounted onto glass, nuclei were stained with 4',6-diamidino-2-phenylindole (DAPI) (Invitrogen, Carlsbad, CA), and the preparations were stored at -20°C before confocal microscopy (Leica TCS SP5 II; Leitz, Heidelberg, Germany).

### Fluorescence-activated cell sorting flow cytometry (FASC)

Vero cells seeded in T-75 flasks containing VP-SFM/4 mM glutamine were allowed to grow to 80% confluence at 37°C. An aliquot of resuspended cells (5 × 10^5^ cells/mL) was mixed with either 80 μg/mL TcdA rRBD or its fragments at 37°C for 5 min. After washing with cold PBST, 1 μg/mL of either the anti-TcdA antibody PCG-4 or an anti-His tag antibody (AbD Serotec, Oxfordshire, UK) was added to the cells, and the mixture was incubated on ice for 30 min. After washing twice with cold PBST, 1 μg/mL FITC-conjugated secondary antibody (Sigma-Aldrich, St. Louis, MO) was added and mixed for surface staining. Before flow cytometry analysis, propidium iodide (Sigma-Aldrich) was added to assess cell viability.

### Hemagglutinin activity analysis

The hemagglutinin (HA) activity assay was performed as described by Wren et al. [37]. In brief, 250 pmol of either TcdA rRBD or its fragments in a 25 μL volume was serially diluted two-fold using PBS buffer in 96-well round-bottom plates. A suspension containing 25 μL of 2% rabbit erythrocytes rewashed with PBS to remove serum contamination was added to the wells at a 1:1 ratio. The mixtures were incubated at 4°C overnight. HA activity was calculated by visual scoring.

### Surface marker and cytokine analyses for DC maturation

DC maturation analysis was performed *in vitro* as previously described [38]. C57BL/6 mice were purchased from the National Animal Center in Taiwan and held in the Animal Center of the NHRI. In brief, bone marrow-derived DCs (BMDCs) were collected from the tibiae of 6- to 8-week old C57BL/6 females. Bone marrow cells were isolated by vigorously washing with lymphocyte-conditioned medium (LCM) (RPMI 1640 containing 1% penicillin and streptomycin, 10% heat-inactivated FBS, 50 μM β-mercaptoethanol, and 50 mM HEPES) and treated with lysis buffer to remove erythrocytes. BMDCs were re-suspended at 2 × 10^6^ cells per mL in LCM and treated with 20 ng/mL recombinant granulocyte macrophage colony stimulating factor (MoGM-CSF) (Peprotech, Rocky Hill, NJ) on days 0 and 3. An aliquot of suspended BMDCs equivalent to 2 × 10^6^ cells/mL was seeded into 24-well plates on day 6. Different concentrations of TcdA rRBD or its fragments with or without 10 ng/mL polymyxin B were added into the wells. LPS extracted from *E*. *coli* O127:B8 (Sigma-Aldrich) and TcdA served as controls. After a 16- to 18-h incubation at 37°C, BMDCs were analyzed by flow cytometry (FACSCalibur, BD Biosciences, Franklin Lakes, NJ) to evaluate the up-regulation of cell surface markers. To exclude immature DCs, which represent 50% of the total cell population, the CD11c^+^ cell population was gated for surface marker staining with specific monoclonal antibodies against CD-40, CD-80, CD-86, and MHC-II. In addition, cell culture supernatants were collected for cytokine expression. Cytokines, such as IL-6, IL-12p40 and TNF-α, were determined using specific cytokine kits purchased from eBioscience (San Diego, CA).

### Circular dichroism (CD) measurement

TcdA rRBD and its fragments (F1, F2 and F3) were prepared in 10 mM phosphate buffer (pH 7.4) at a concentration of 50 μg/mL. Spectra were obtained with a J-185 spectropolarimeter (Jasco, Easton, MD) with thermal electric temperature control, and the data were acquired in continuous scanning mode with a 0.5 mm path length, a 1 nm interval and an accumulation time of 10–15 s/min. The far UV scan range was set between 260 and 200 nm with a scan speed of 50 nm/min. All data were processed using Jasco software. The background spectrum obtained with 10 mM phosphate buffer (pH 7.4) was subtracted from the acquired sample spectrum for the thermal stability test, and the temperature gradient was set between 30°C and 90°C. CD spectra were recorded at intervals of 10°C. The percent of each secondary structure was calculated from the mean residue ellipticity ([θ]) according to the method suggested by the manufacturer.

### Mouse immunogenicity study

BALB/c mice were purchased from the National Animal Center in Taiwan and held in the Animal Center of the NHRI. Eight groups of mice (6 BALB/c mice per group) were vaccinated with three intramuscular (IM) injections of 3, 10 and 30 μg of either TcdA rRBD or truncated fragments (F1, F2 or F3) with and without alum (AlPO_4_) every two weeks. Before each immunization, mice were bled to collect sera, which were stored at -20°C, for anti-RBD antibody titer determination in an RBD-specific ELISA. To study the adjuvant effects of TcdA rRBD, individual groups of 6 BALB/c mice were immunized intramuscularly with 2 μg of ovalbumin (OVA) (Sigma-Aldrich) formulated with 0.3, 3 or 10 μg of either TcdA rRBD or aluminum phosphate (AlPO_4_) (Sigma-Aldrich). Animals that received 2 μg of OVA alone served as the controls. The mice were given three immunizations at two-week intervals and bled before each injection. Sera were collected and stored at -20°C for anti-OVA antibody titer measurement using an OVA-specific ELISA as described below.

### Antigen-specific ELISA

ELISA plate wells were coated with 100 ng of either TcdA rRBD or OVA overnight and then blocked with 5% nonfat dry milk (w/v) in PBS. Mouse antisera serially diluted 2-fold with PBS containing 1% BSA (Calbiochem, Darmstadt, Germany) were added to the wells, followed by incubation at room temperature (RT) for 2 h. Each group of pre-immunized serum was diluted fifty-fold with PBS. After washing three times with PBST, 50 ng/mL of either anti-IgG isotypes (Invitrogen, Carlsbad, CA) or anti-IgA (Invitrogen, Carlsbad, CA) HRP-conjugated IgG (KPL, Gaithersburg, MD)-specific antibodies diluted in PBS containing 1% BSA was added to the wells and incubated at RT for 1 h. After washing three times with PBST, the plates were treated with TMB peroxidase substrate (KPL) at RT in the dark for 20 min. To determine the anti-RBD or anti-OVA titers, the OD_450 nm_ absorbance was measured using a spectrophotometer.

### Anti-toxin neutralization assay

The anti-TcdA neutralization assay was performed according to the protocol previously described by Seregin *et al*. [23]. Briefly, Vero cells (2 × 10^4^ per well) were seeded into 96-well plates containing VP-SFM culture medium and 4 mM glutamine at 37°C, and the cells were allowed to grow to confluence. Mouse sera from mice immunized either with TcdA rRBD or truncated fragments with and without alum were serially diluted two-fold with fresh VP-SFM, mixed with an equal volume of 20 ng/mL TcdA from *C*. *difficile* VPI 10463 (The Native Antigen Company Ltd, Oxfordshire, UK) and incubated at room temperature for 1 h. The mixture was added to the 96-well plates containing Vero cells and incubated at 37°C for 24 h. Anti-toxin neutralization titers were calculated as the highest serum dilution that could protect 50% of the cells from rounding due to toxin cytotoxicity. Cellular toxicity was recorded using a microscope equipped with a camera.

### Toxin A challenge mouse model

A lethal TcdA challenge mouse model was established to assess the efficacy of anti-RBD immune responses *in vivo* using the protocol previously described by Seregin et al. [23]. Briefly, 4 groups of BALB/c mice (10 mice per group) were immunized IM with either PBS or three dosages of TcdA rRBD (0.3, 3, and 30 μg) at days 0, 14, and 28. After three immunizations, mice were challenged with 150 ng of TcdA from *C*. *difficile* VPI 10463 (5× lethal dose (LD_50_)) (The Native Antigen Company) by intra-peritoneal injection at day 35, and the survival rate was monitored for 14 days. The mice were observed twice daily during the first 3 days.

### Statistical analysis

The data were expressed using Prism 5 version 5.01 (GraphPad Software, Inc.). The antibody titers are displayed as the mean±SEM from the experiments. Significant differences were analyzed using a two-tailed Student’s t-test comparing the means obtained for each treatment with the control group. A *p*-value of <0.05 was considered significant.

## Results

### Design of the consensus sequence of RBD

Based on previous studies [8, 22, 24, 29–30], the receptor-binding domain of TcdA is located between residues 1800 and 2710 in its C-terminal region and has a molecular size of approximately 100 kDa. Amino acid sequences of TcdA RBD from different *C*. *difficile* isolates deposited in the NCBI protein database were aligned and examined. The results indicated that the TcdA RBD amino acid sequence was highly conserved with 97% identity. Moreover, the amino acid sequences of the RBDs from different *C*. *difficile* isolates could be divided into two groups. One group was biased to strains including VPI 10463 and ATCC9689, which are the reference strains for *C*. *difficile* toxin studies, and the other group included the majority of PCR-ribotype 027 isolates. In this study, the selected consensus amino acid sequence of RBD was composed of 911 residues and was identical to that of BI/NAP1/027-related isolates, as expected because the majority of the protein sequences deposited in the NCBI database are ribotype 027 strains (Fig 1A). The differences between the amino acids of the consensus sequence and the VPI 10463 reference strain are highlighted in Fig 1A. There are 33 synonymous changes within the RBD, including 11, 24 and 16 amino acids that differ in the F1, F2 and F3 fragments, respectively. In addition, 12 changes were found within the 7 potential lectin-binding sites. These differences affected the cell-binding activities of these fragments and influenced the quality of the antibodies generated in the animal immunogenicity studies (see below). Upon further sequence analysis, the consensus sequence contained 34 short repeat units containing 18 to 22 amino acids (Fig 1B). Previous studies by Dove *et al*. [30] and Ho *et al*. [31] suggested that the RBD contained 38 and 32 short repeat units, respectively. This discrepancy in the estimated number of repetitive sequences may be the result of using different analysis software programs.

To study the specific roles and functions of the 7 putative receptor-binding regions, TcdA RBD was divided into three fragments, F1, F2 and F3, which correspond to the N-terminal residues 1 to 411, the middle region residues 296 to 701, and the C-terminal residues 524 to 911 of TcdA RBD, respectively (Fig 1C). Each fragment was purposely designed to contain 3 potential lectin-like (oligosaccharide) receptor-binding (LR) sites. F1 and F2 contain an overlapping LR site #3; F2 and F3 have an overlapping LR site #5 (Fig 1C).

### Production of recombinant RBD (rRBD) and its fragments

**Fig 2:**
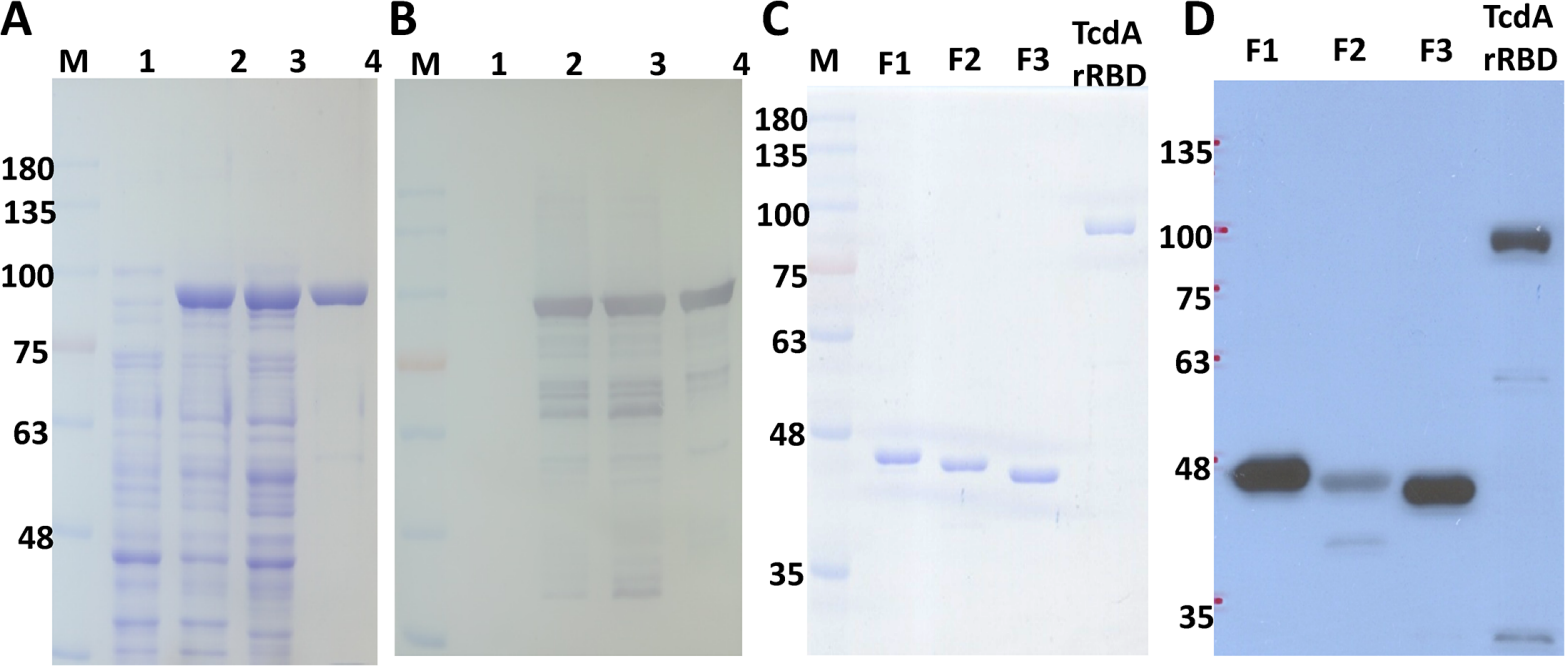
The biochemical characterization of recombinant *C*. *difficile* TcdA rRBD. TcdA rRBD and its truncated fragments with the consensus sequence expressed in the *E*. *coli* JM109 strain. The expression and purification of TcdA rRBD were confirmed by SDS-PAGE (A) and western blot with a toxin A-specific monoclonal antibody (B); lanes 1 to 4 represent non-induction, induction, crude lysate, and chromatographic purified samples, respectively. The purity of TcdA rRBD fragments F1, F2 and F3 was confirmed by SDS-PAGE (C) and western blot with a toxin A-specific monoclonal antibody (D).

The DNA coding sequence of the tcdA RBD was designed employing codon usage optimization, synthesized, inserted into the pET-22b vector and successfully expressed in *E*. *coli* as shown in Fig 2. After a single-step purification using Ni-affinity chromatography, highly purified TcdA rRBD was obtained, and its purity was analyzed by SDS-PAGE to be >95% (Fig 2A); trace amounts of TcdA rRBD degradation fragments were detected using a TcdA-specific antibody by western blot analysis (Fig 2B). These degradation products are likely the result of proteolytic digestion during the purification process [39]. In any event, at least 20 mg of highly enriched TcdA rRBD (Fig 2B, lane 4) was easily obtained from 1 L of bacterial culture. Most of the *E*. *coli* endotoxin (LPS) was successfully removed by passing the TcdA rRBD preparation through an E membrane; the residual LPS in the purified TcdA rRBD was found to be below 30 EU per mL based on the LAL assay. Although a simple and rapid method for producing TcdA rRBD with high purity was successfully developed, TcdA rRBD was found to be unstable and easily lost its biological function during the freeze-thaw process (see below). We found that the best conditions for preserving TcdA rRBD integrity were to store the protein at 3 to 5 mg/mL in PBS containing 10% (v/v) glycerol at -80°C.

Using the same rapid purification method, highly purified F1 (25 mg), F2 (15 mg) and F3 (50 mg) fragments of TcdA rRBD were successfully obtained from 1 L of bacterial culture. The purity was >97%, as analyzed by SDS-PAGE (Fig 2C) and western blot analysis using a TcdA-specific monoclonal antibody (Fig 2D). The residual LPS in the purified F1, F2 and F3 preps were all <10 EU per mg of protein based on the LAL assay. These recombinant RBD fragments were also stored in PBS containing 10% (v/v) glycerol at -80°C. F1 and F3 had the same solubility as TcdA rRBD (>3 mg/mL), but F2 was less soluble and was stored at <1 mg/mL. As shown in Fig 2D, F2 was not well recognized by the anti-TcdA monoclonal antibody PCG-4, which was generated from the VPI 10463 strain. This lower antigenicity may be attributable to the 24 amino acid changes in F2 compared to those in the VPI 10463 strain (Fig 1A).

### Biological characterization of rRBD and its fragments

CD secondary structure analysis of TcdA rRBD and its fragments was performed to confirm that the proteins were correctly folded to form a majority β-sheet structure (>43%) (S1A Fig). The results are consistent with other reports indicating that the fragments of RBD containing 5 to 15 short repetitive sequences could form stable folded β-solenoid secondary structures independent of other functional domains in the TcdA [31–32]. In addition, the thermal stability of each recombinant protein was determined, and the melting temperatures (Tm) for TcdA rRBD, F1, F2 and F3 were found to be 48.5, 43.8, 47.3 and 51.5°C, respectively (S1B Fig).

**Fig 3:**
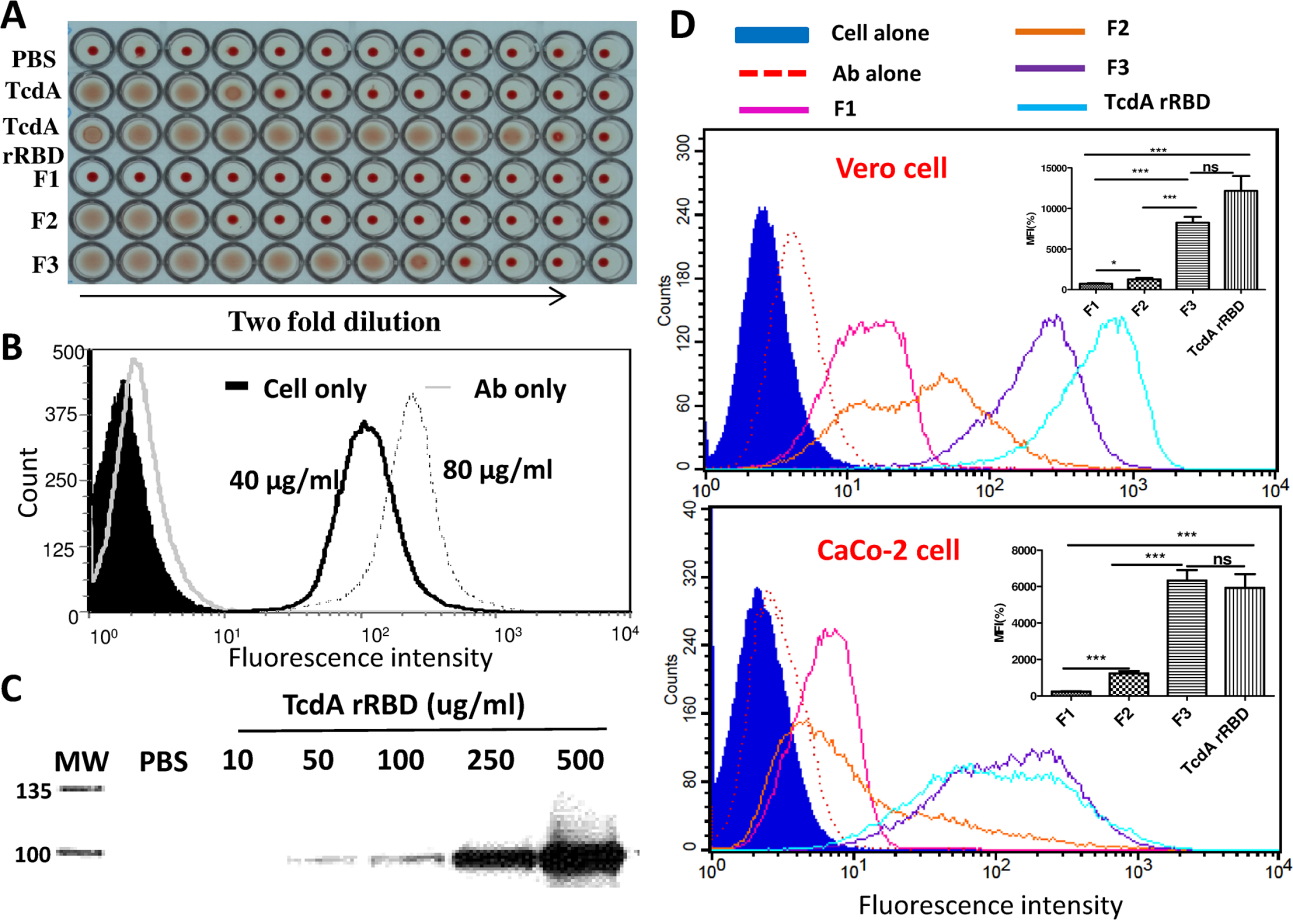
Several functional assays were used to evaluate the biological properties of TcdA rRBD and its fragments. (A) A hemagglutinin (HA) assay was performed starting with either 15 pmol of TcdA or 250 pmol of TcdA rRBD, F1, F2 and F3 mixed with 2% rabbit erythrocytes in a 1-to-1 ratio (v/v). TcdA rRBD cell-binding ability was characterized by flow cytometry (B) and western blot (C) using an anti-TcdA specific monoclonal antibody. Different amounts of TcdA rRBD were used to treat cells to evaluate binding ability in a dose-dependent manner. (D) Vero and Caco-2 cell-binding abilities of TcdA rRBD and its fragments (1 μM) were characterized by FACS analysis as described in the Materials and Methods. The MFI from TcdA rRBD fragment binding assays were measured to evaluate statistical significance, as shown in the bar charts within the FACS figures. The symbols *, ** and *** indicate *p*<0.05, *p*<0.01 and *p*<0.005, respectively.

The hemagglutinin activity of TcdA rRBD was evaluated using rabbit erythrocytes. TcdA rRBD could agglutinate rabbit erythrocytes at concentrations as low as 0.4 pmol (Fig 3A) and was more potent than TcdA (1.6 pmol). The biological function (HA activity and binding to Vero cells) of TcdA rRBD correlates with its active conformation because freeze-thaw and/or boiling processes either reduced or eliminated these biological properties. Therefore, preserving the functionally active conformation of TcdA rRBD is very important; however, it is easily influenced by purification and storage conditions. Together, these results indicate that the purified TcdA rRBD is correctly folded and exhibits functions similar to those of the receptor-binding domain of native toxin A.

To investigate whether TcdA rRBD fragments possess hemagglutinin activity, F1, F2 and F3 were evaluated for HA activity using rabbit erythrocytes. As shown in Fig 3A, F3 had similar HA activity to TcdA (2 pmol), while F2 (32 pmol) had much lower HA activity (p<0.01). Surprisingly, F1 did not agglutinate rabbit erythrocytes even at protein concentrations as high as 1 μmol (Fig 3A). However, the β-sheet secondary structure revealed by the CD spectra analysis indicated that the purified F1 folded correctly (S1 Fig).

To confirm the biological function of TcdA rRBD, Vero cell-binding activity was analyzed by western blot and flow cytometry. The results shown in Fig 3B indicate that 0.4 μM (40 μg/mL) TcdA rRBD was unambiguously bound to the Vero cell surface, as detected by FACS analysis. We also demonstrated that TcdA rRBD was able to recognize and strongly bind to Vero cells in a dose-dependent manner, as assessed by both FACS and western blot analyses (Fig 3B and 3C). The binding of TcdA rRBD fragments to Vero cells was tested using FACS analysis. At 1 μM protein concentration, F3 had the highest fluorescence intensity, as shown in Fig 3D. F1 had the lowest binding and fluorescence intensity compared to TcdA rRBD and F3. F2 had bi-phasic binding activity to Vero cells, indicating that F2 binds to at least two types of receptors. As shown in Fig 2D, the TcdA-specific monoclonal antibody recognized F2 least; thus, to eliminate the reduced antibody binding affinity, FACS analyses were repeated with an anti-His tag antibody, and similar results were obtained (data not shown). This result confirmed that the binding activity to Vero cells was as follows: TcdA rRBD>F3>F2>F1 (*p*<0.001). To further show the sensitivity of Vero cell binding, different concentrations of TcdA rRBD and its fragments were tested. Under the current binding assay conditions, the minimum concentration required to induce a 3-fold increase in fluorescence intensity compared to the negative control (ovalbumin) for TcdA rRBD, F1, F2 and F3 bound to Vero cells was 10, 50, 500, and 10 nM, respectively.

The binding of TcdA rRBD and its truncated fragments to Caco-2 cells was also evaluated using FACS analysis. At the 1 μM protein concentration, TcdA rRBD and F3 exhibited the highest binding and fluorescence intensity to Caco-2 cells, as shown in Fig 3D, but the bi-phasic fluorescence intensity suggested that both rRBD and F3 could bind at least two types of receptors. F1 and F2 exhibited lower binding and fluorescence intensity compared to TcdA rRBD and F3. F2 also showed multi-phasic binding activity to Caco-2 cells, indicating that F2 binds to more than two types of receptors. As shown in Fig 3D, the current results indicate that the binding activity to Caco-2 cells was as follows: F3≥TcdA rRBD>F2≥F1 (*p*<0.001).

**Fig 4:**
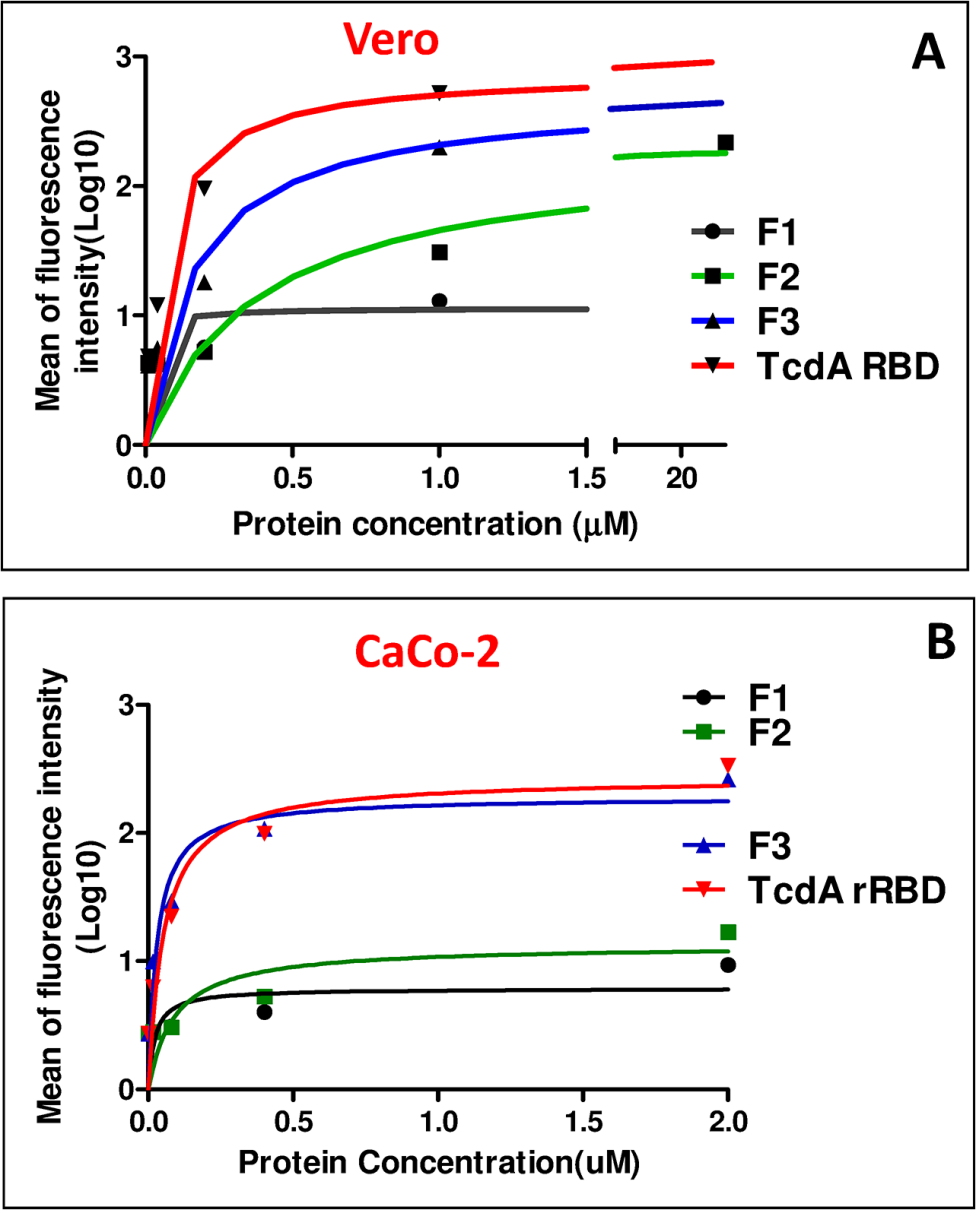
Cell-binding affinities and kinetics of TcdA rRBD and its fragments were determined by FACS analysis as described in the Materials and Methods.

The binding affinity and kinetics of TcdA rRBD and its fragments to Vero cells were further analyzed, and the apparent K_d_ (defined as the half-maximal binding to the receptors) for TcdA rRBD was 0.14 μM (Fig 4A). The results from the fragment cell-binding studies indicate the apparent K_d_ values to be 0.26, 0.56 and 0.16 μM for F1, F2 and F3, respectively (Fig 4A). TcdA rRBD and F3 quickly bound to certain receptor(s) on the surface of Vero cells, and >8 μM TcdA rRBD and F3 were required to saturate all receptors (Fig 4A). In contrast, the binding of F1 to its receptor(s) was rapid and saturated at 2 μM. These results suggest that F3 and TcdA rRBD have similar binding activities but may have different binding specificities and sensitivities compared to F1.

The binding assay was also performed with Caco-2 cells, and the apparent K_d_ was 0.09 μM for TcdA rRBD, which was similar to that obtained from the Vero cell studies (Fig 4B). The apparent K_d_ values were 0.18, 0.32 and 0.08 μM for F1, F2 and F3, respectively (Fig 4B). F3, similar to TcdA rRBD, binds to Caco-2 cells much stronger and more rapidly than F1 and F2 (p<0.001). All cell binding was easily saturated at 1 μM (Fig 4B). These results show that TcdA rRBD and F3 have similar binding specificities and sensitivities to Vero and Caco-2 cells but differ from F1.

To optimize the protein concentration used for the identification of surface receptor(s) in Vero cells, F1 cell binding was analyzed by immunoblot. F1 bound strongly to Vero cells in a dose-dependent manner, as analyzed by immunoblotting (S2 Fig). In the same assay, both F2 and F3 exhibited less binding than F1 (S2 Fig). These immunoblot results are inconsistent with those obtained from flow cytometry, in which F3 demonstrated significantly better binding (*p*<0.001) than F1 to the receptor(s) on the surface of Vero cells (Fig 3D). This discrepancy could be attributable to the 30-min cell binding incubation time, in which most F3 translocated into the cytosol but F1 remained bound to the receptor on the cell surface (see the cellular uptake of TcdA rRBD and its fragment below). Therefore, much less F3 was observed in the immunoblot analysis of cell membrane lysates. Preliminary results indicate that 30 to 60 μg of F1 are sufficient to form strong complexes with the cell receptors, which could be pulled down with the anti-TcdA monoclonal antibody (Huang *et al*., unpublished result).

### Cellular uptake of TcdA rRBD and its fragments

**Fig 5:**
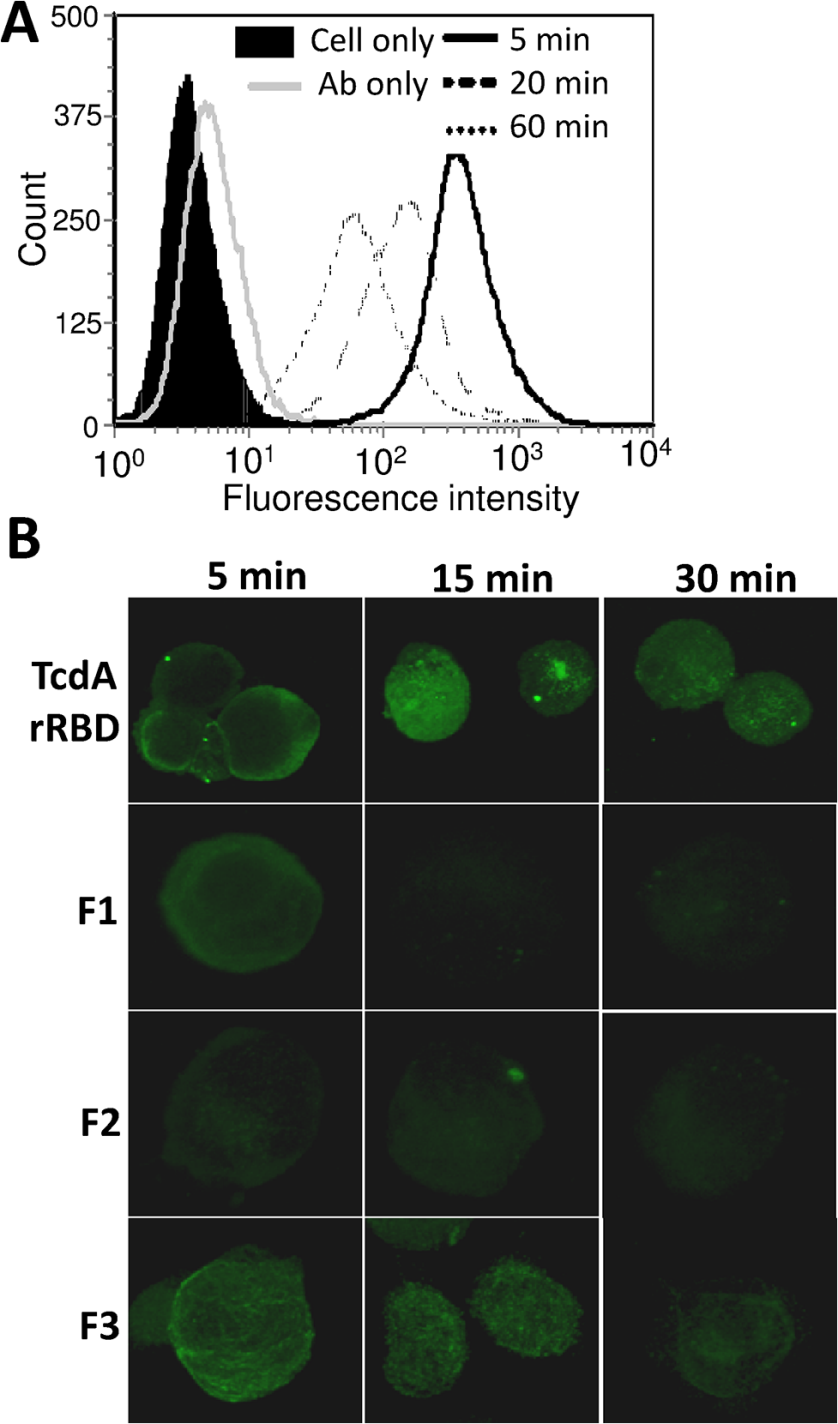
Cellular uptake of TcdA rRBD and its fragments. (A) TcdA rRBD binding signals on the surface of Vero cells were detected by flow cytometry after TcdA rRBD inoculation at the indicated times. (B) The internalization signals of TcdA rRBD and its fragments in Vero cells were evaluated by confocal microscopy at 5, 15, and 30 min. The images were collected from a single stack in the central region of the *z*-axis. Green fluorescence signals represent the locations of TcdA rRBD, F1, F2 and F3.

*C*. *difficile* toxin binding to cell surfaces promotes its cellular internalization through receptor-mediated clathrin-dependent endocytosis [40–41]. To verify whether TcdA rRBD exhibits this biological activity, a Vero cell-binding assay was performed *in vitro*, and TcdA rRBD binding was analyzed by flow cytometry. After TcdA rRBD binding, the specific immunofluorescence signal against TcdA rRBD decreased with time (Fig 5A). These decreases in fluorescence signal intensity suggest that TcdA rRBD could either be internalized into the cytosol from the surface or degraded. Therefore, confocal immunofluorescence assays were performed to localize TcdA rRBD after binding to Vero cells. The results indicate that the cellular uptake of TcdA rRBD occurs very quickly (Fig 5B). The cell surface-binding and internalization processes of TcdA rRBD were observed after 5 min of incubation, as illustrated by strong anti-rRBD fluorescence signals around the cell surface. After 15 min of incubation, the majority of TcdA rRBD had entered the cytosol, as indicated by very strong confocal immunofluorescence signals (Fig 5B). After 30 min, a decrease in the immunofluorescence signal was noted and was likely attributable to lysosomal degradation (Fig 5B). These trends support the notion that TcdA rRBD binds specifically to the cell surface, is internalized into the cytosol, and is cleaved into fragments by lysosomal degradation. Due to its rapid internalization process, rRBD could potentially serve as a carrier for drug delivery systems.

Because F1, F2 and F3 are all capable of binding to Vero cells, it is of interest to determine whether they would be internalized into the cytosol after binding to the cell receptor(s). As illustrated by immunofluorescence signals in the confocal studies, cell surface binding and internalization processes were observed for F2 and F3 after 15 and 5 min of incubation, respectively (Fig 5B). After 15 min of incubation, strong confocal immunofluorescence signals suggested that F3 entered the cytosol. Similar to TcdA rRBD, a decrease in the immunofluorescence signal was observed after 30 min of incubation (Fig 5B). These findings support the notion that F3 specifically binds to the cell surface, is internalized into the cytosol, and is cleaved into fragments by lysosomal degradation. After a 15-min incubation, F2 exhibited weak confocal immunofluorescence signals, which suggested that some F2 entered the cytosol (Fig 5B). Conversely, after 30 min of incubation (and even after 75 min), F1 exhibited very weak confocal immunofluorescence signals, which suggested that little F1 entered the cytosol (Fig 5B). To confirm that the cellular uptake was receptor-mediated clathrin-dependent endocytosis, chlorpromazine (CPZ) was used and found to inhibit TcdA rRBD and F3 internalization, as shown in S3 Fig. Based on the results obtained from the cell-binding studies, HA assay and cellular uptake studies, the putative receptor-binding domains in F1, F2, and F3 likely play different and specific biological roles and functions.

### Dendritic cell maturation triggered by TcdA rRBD

**Fig 6:**
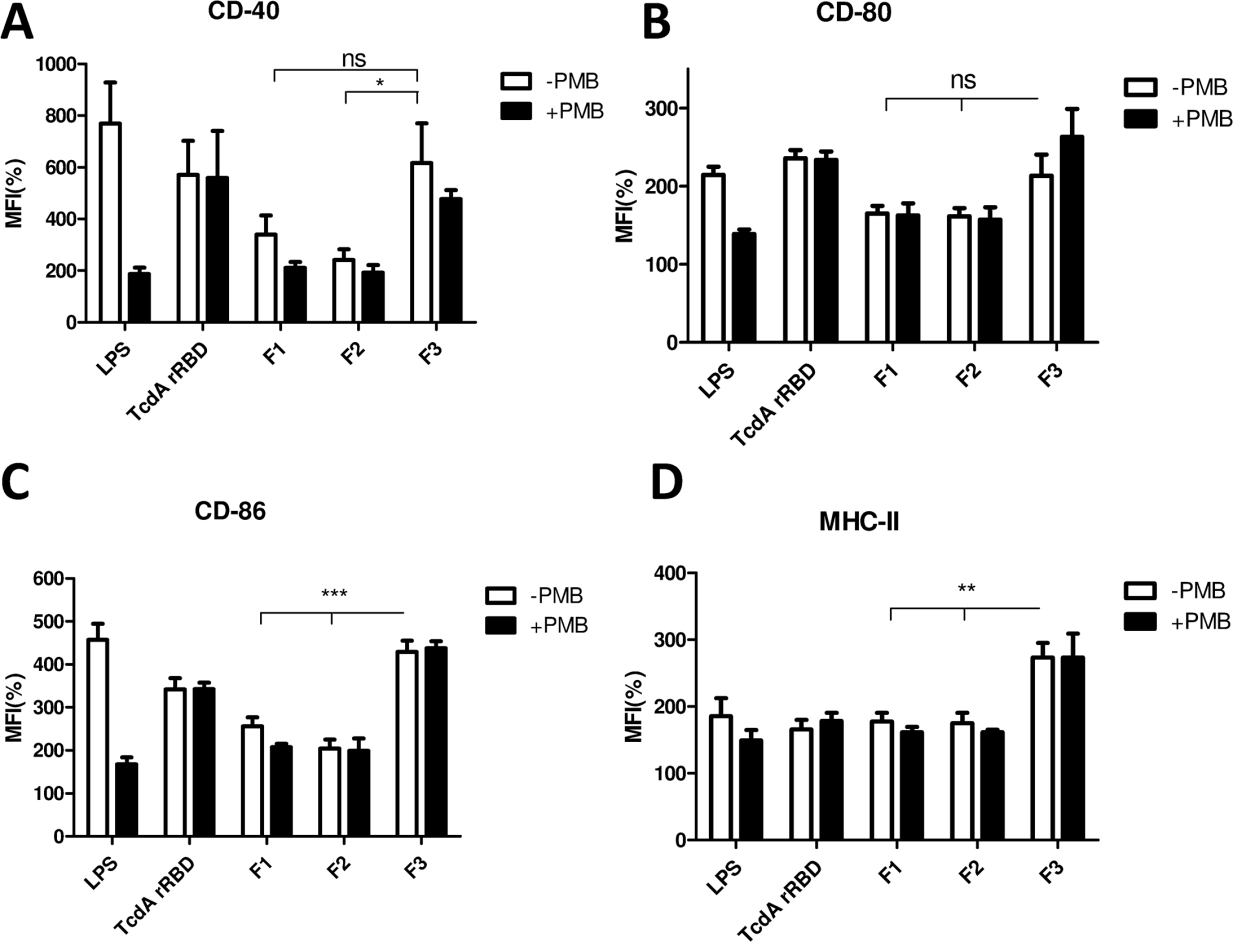
Up-regulation of surface biomarkers of BMDCs by either TcdA rRBD or its truncated fragments. BMDCs from C57BL/6 were collected and treated with GM-CSF at days 0 and 3. TcdA rRBD was added at day 6 for 18 h, and then, DCs were collected to analyze their surface markers, including CD40 (A), CD80 (B), CD86 (C), and MHC II (D), by flow cytometry. All groups were divided as polymyxin B-treated (PMB) (black-net bar) or non-treated (gray-net bar) to exclude LPS contamination. All surface marker signaling was normalized by calculating the ratio of mean fluorescence intensity (MFI) between the medium control and treatments. The symbols *, ** and ns indicate *p*<0.05, *p*<0.01 and no significant difference, respectively.

**Fig 7:**
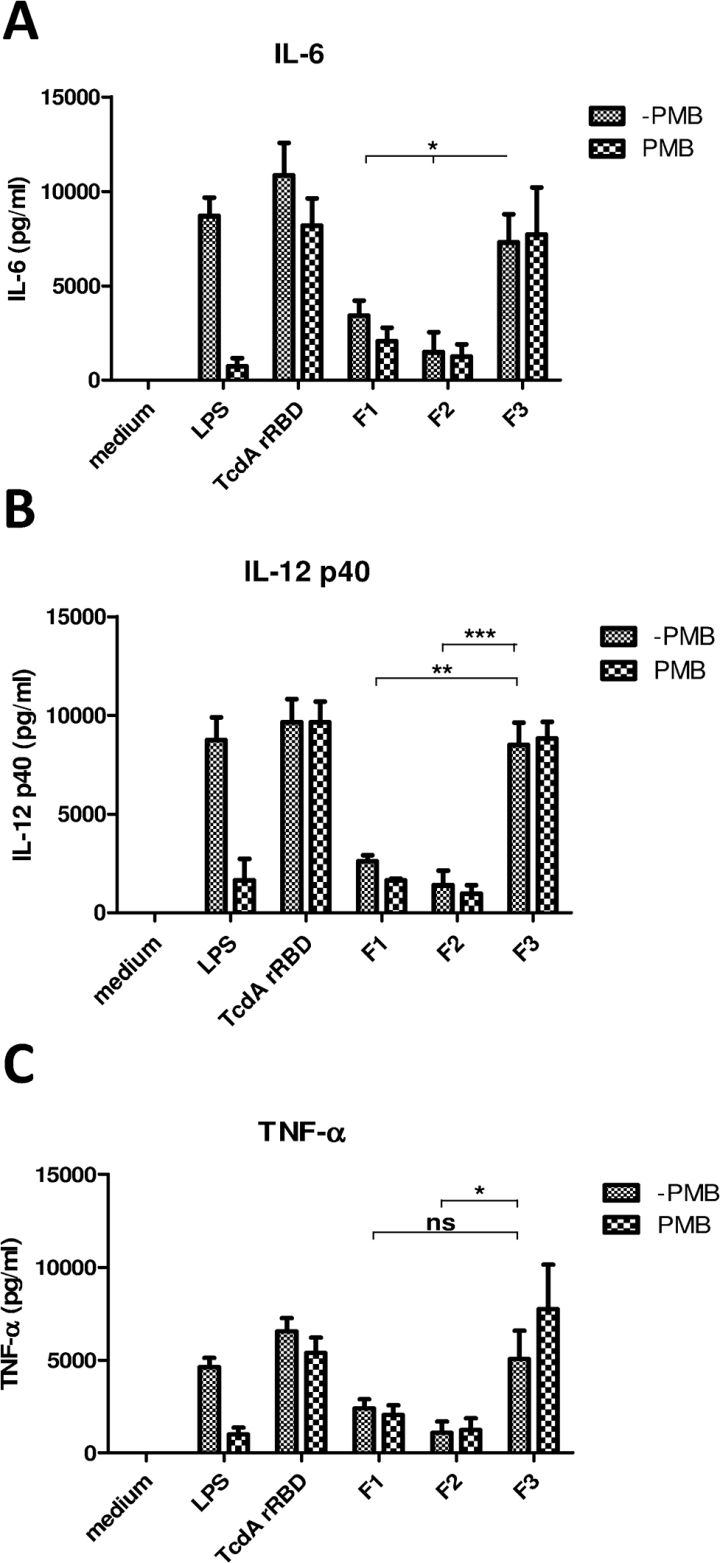
Cytokine secretion from BMDCs treated with TcdA rRBD or its truncated fragments. After BMDCs were treated with TcdA rRBD or its truncated fragments at day 6 for 18 h, the culture supernatants were collected and analyzed for cytokine profiles using specific cytokine ELISAs: (A) IL-6, (B) IL12p40, and (C) TNF- α. The symbols * and ** indicate *p*<0.05 and *p*<0.01, respectively.

*C*. *difficile* toxin A has been reported to up-regulate cell surface marker expression and cytokine secretion from dendritic cells [42]. In addition, previous studies [43–44] have reported that the C-terminal part of TcdA could serve as a mucosal adjuvant to enhance the immune response toward a co-administered antigen. Therefore, TcdA rRBD was tested for its ability to promote the maturation of DCs. BMDCs from C57BL/6 mice were treated with increasing amounts (0.3 to 10 μg) of TcdA rRBD. Cell surface biomarkers associated with DC maturation (CD-40, CD-80, CD-86, and MHC-II) and the secretion of pro-inflammatory cytokines (IL-6, IL12, and TNF-α) were examined using FACS analysis and cytokine-specific ELISA, respectively. To preclude the interference of LPS contamination, TcdA rRBD samples used in the current studies had very little LPS contamination (0.03 EU/μg of protein). In addition, polymyxin B was added to the DC samples to prevent activation by LPS through the Toll-like receptor 4 pathway. Surface biomarkers of DC maturation were up-regulated, and the production of pro-inflammatory cytokines (IL-6, IL12, and TNF-α) increased in a dose-dependent manner from 0.5 to 6 μM (data not shown). A 2-μM TcdA rRBD concentration in the final assay solution was selected for subsequent analyses. Both DC maturation biomarkers were up-regulated (Fig 6A to 6D), and a significant increase in the production of pro-inflammatory cytokines (IL-6, IL12, and TNF-α) was detected in TcdA rRBD-treated BMDC culture supernatant (Fig 7A to 7C). The results were not influenced by minor LPS contamination, as there was no significant difference between polymyxin B-treated and non-treated samples, as shown in Figs 6 and 7. We boiled both TcdA rRBD samples and LPS to denature and destroy their biological functions. Boiling did not affect LPS-induced DC activation but fully abolished TcdA rRBD-mediated DC activation. Overall, this result clearly demonstrates that DC activation is mediated by TcdA rRBD.

To assess whether the fragments of TcdA rRBD have similar adjuvant properties to TcdA rRBD, both DC maturation biomarker and the production of pro-inflammatory cytokine (IL-6, IL12, and TNF-α) assays were performed with 2 μM of F1, F2 or F3 with and without polymyxin B. DC biomarkers and the production of pro-inflammatory cytokines were up-regulated and increased in BMDC culture (Figs 6 and 7). Interestingly, as shown in Figs 6 and 7, F1 and F2 were less effective (p<0.01) in inducing DC maturation biomarkers and the production of pro-inflammatory cytokines (IL-6, IL12, and TNF-α) compared to cells treated with either TcdA rRBD or F3. These results are consistent with previous reports [42–44] and suggest that TcdA rRBD and F3 are potential candidates for recombinant subunit vaccine development against CDI and can be used as potent adjuvants to enhance immune responses against weak immunogens (see below).

### Mouse immunogenicity studies

To assess the immunogenicity of TcdA rRBD, groups of mice were vaccinated with either TcdA rRBD alone or formulated with alum (AlPO4). Analyses of antisera from mice immunized with varying doses of TcdA rRBD using a RBD-specific ELISA revealed that TcdA rRBD alone could induce significant antibody responses (S4A Fig). The results from the current mouse immunogenicity studies indicate that TcdA rRBD is highly immunogenic because mice vaccinated twice with 3 μg of TcdA rRBD alone produced very strong anti-rRBD IgG antibodies with a titer of >10^4^ (week 4 in S4A Fig). Moreover, in antisera from mice vaccinated 3 times with TcdA rRBD, both IgG_1_ and IgG_2_ isotype antibody responses were observed (S4B Fig). Systemic IgA antibody responses were also elicited by TcdA rRBD vaccination (S4B Fig). The pre-immunized mouse sera and/or sera from mouse immunized with PBS served as controls, and no anti-RBD IgA antibody signal was detected at fifty-fold dilution. At 6 weeks post-vaccination, the anti-rRBD IgG antibody titers (~ 1 × 10^5^) elicited by 3 μg of TcdA rRBD alone were not different from those obtained with either 3 × 10 μg of TcdA rRBD formulated with alum or 3 × 10 μg of TcdA toxoid (S4A Fig and Table 1). These results clearly indicate that 3 μg of TcdA rRBD can induce strong anti-rRBD IgG antibody responses. The anti-rRBD IgG antibody titers (~ 1 × 10^5^) could be the maximal antibody responses elicited by TcdA rRBD because 30 μg of TcdA rRBD did not enhance the titer (5 × 10^4^) (S4A Fig and Table 1).

**Table 1:** *C*. *difficile* toxin A neutralization titers of antisera from groups of 6 mice immunized 3 times with varying amounts of either TcdA rRBD or its fragments formulated with or without alum.

Mice immunized with		Anti-rRBDIgG titers^a^	Neutralization titers^b^	Protection rate (%)^c^
TcdA rRBD	0 μg	<100	<4	0
	3 μg	9.3 × 10^4^	32	16
	10 μg	1.7 × 10^5^	256	ND
	30 μg	5.3 × 10^4^	128	67
	10 μg + alum	1.2 × 10^5^	128	83
RBD F1	10 μg	1.7 × 10^4^	8	ND
	10 μg + alum	1.5 × 10^6^	64	50
RBD F2	10 μg	6.7 × 10^3^	4	ND
	10 μg + alum	6.5 × 10^4^	16	0
RBD F3	10 μg	1.1 × 10^4^	16	ND
	10 μg + alum	8.1 × 10^4^	64	50
TcdA toxoid^d^	10 μg	7.7 × 10^4^	256	ND
	10 μg + alum	3.7 × 10^5^	2048	100

^a^ The titers were obtained from sera pooled from 6 mice.

^b^ The neutralization titer against toxins was defined as the highest sample dilution that could prevent 50% of cell rounding induced by toxins.

^c^ Groups of 6 mice were immunized 3 times with 10 μg of different RBD proteins formulated with or without alum and then challenged with 5 × LD_50_ of toxin A. The survival rate in each group was reported at 36 h post-challenge.

^d^ A group of 6 mice was immunized 3 times with 10 μg of TcdA toxoid formulated with or without alum.

ND indicates not performed.

**Fig 8:**
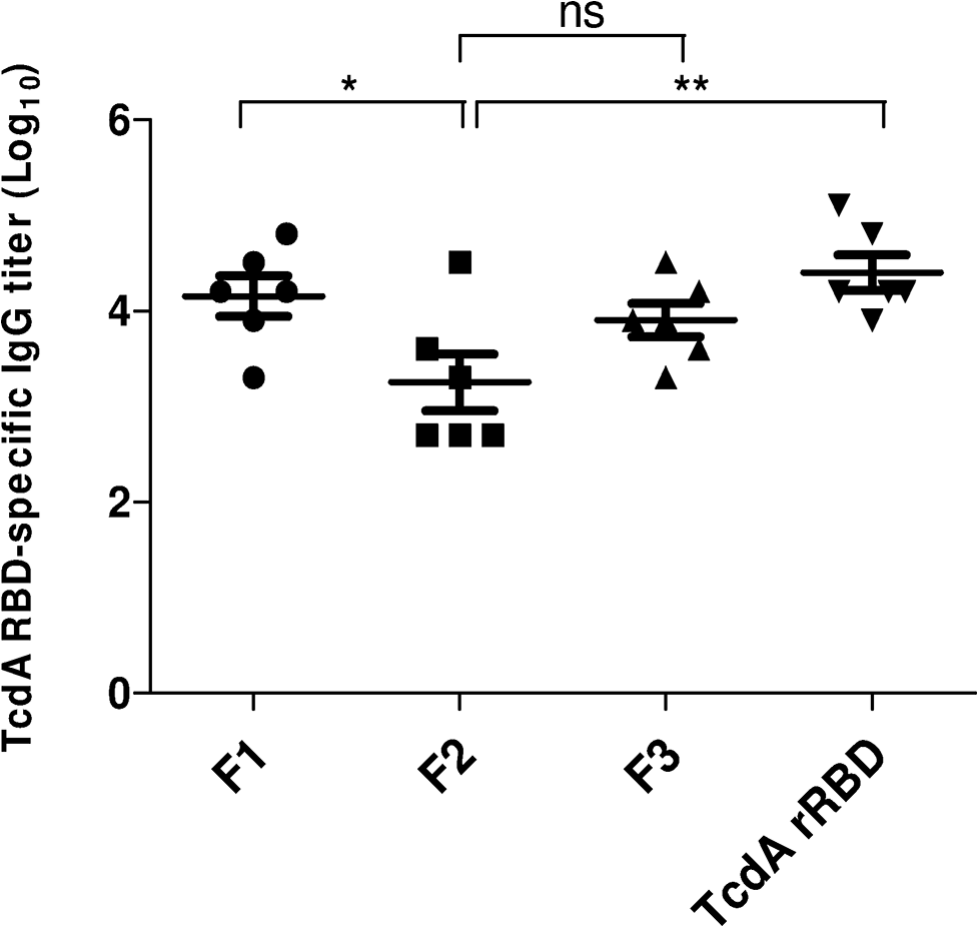
The immunogenicity of TcdA rRBD and its truncated fragments in mice. BALB/c mouse anti-RBD antibody responses elicited by 3 × 10 μg of either TcdA rRBD or its fragments. Anti-RBD IgG titers at 6 weeks were determined by RBD-specific ELISA.

When mouse immunogenicity was assessed with each RBD fragment, the 6-week post-vaccination anti-RBD IgG antibody titers (~ 1 × 10^4^) elicited by 10 μg of either F1 or F3 were not different from those obtained with 10 μg of TcdA rRBD (Fig 8). Antisera from mice vaccinated three times with 10 μg of F2 had lower levels of anti-rRBD IgG antibodies compared with those obtained from either F1 (*p*<0.01) or F3 (*p*<0.05). Interestingly, anti-rRBD IgG antibody titers were enhanced by 5- to 10-fold when the RBD fragments were formulated with alum, but that result was not observed with 3 × 10 μg of TcdA rRBD formulated with alum (S5 Fig and Table 1). As shown in S5 Fig, alum formulated with F1 elicited the strongest anti-RBD antibody responses compared to those obtained from alum formulated with either F2 (*p*<0.001) or F3 (*p*<0.01).

### Immunological characterization of anti-rRBD sera

To test whether mouse anti-RBD antibodies elicited by TcdA rRBD could functionally neutralize the cytotoxicity of TcdA, antisera were evaluated in a Vero cell cytotoxicity assay as described in the **Materials and Methods**. As shown in Table 1, antisera from mice immunized with 10 μg of TcdA rRBD were found to have a neutralization titer (NT) equal to 256 that prevents 50% of the cell death resulting from toxin A cytotoxicity. The neutralization titers obtained from mice immunized with 3 × 10 μg of TcdA rRBD formulated with alum were insignificant compared to those obtained from TcdA rRBD alone (Table 1). Surprisingly, the mouse neutralization titer (NT = 128) obtained with 30 μg of rRBD without adjuvant was comparable to titers elicited by 10 μg of TcdA rRBD alone (NT = 256) or formulated with alum (NT = 128) or 10 μg of TcdA toxoid alone (NT = 256) (Table 1). In contrast, the highest anti-TcdA NT was obtained from mice immunized with 3 × 10 μg of TcdA toxoid formulated with alum (NT = 2048). The current results indicate that 10 μg of TcdA rRBD alone could induce significant functional neutralizing antibody levels against TcdA. The anti-RBD IgG antibody responses elicited by freeze-thaw or heat-treated rRBD were significantly lower and exhibited no neutralizing activity (data not shown). Thus, preserving the functionally active conformation of TcdA rRBD is very important.

To test whether antisera obtained from mice vaccinated 3 times with 10 μg of F1, F2 or F3 alone could functionally neutralize the cytotoxicity of TcdA, antisera were assessed in the Vero cell cytotoxicity assay and had low NT (NT = 8, 4 and 16 for F1, F2 and F3, respectively) (Table 1). In contrast, the neutralization titers were significantly (p<0.05) increased (at least 4-fold) when the mice were immunized with RBD fragments formulated with alum (Table 1).

**Fig 9:**
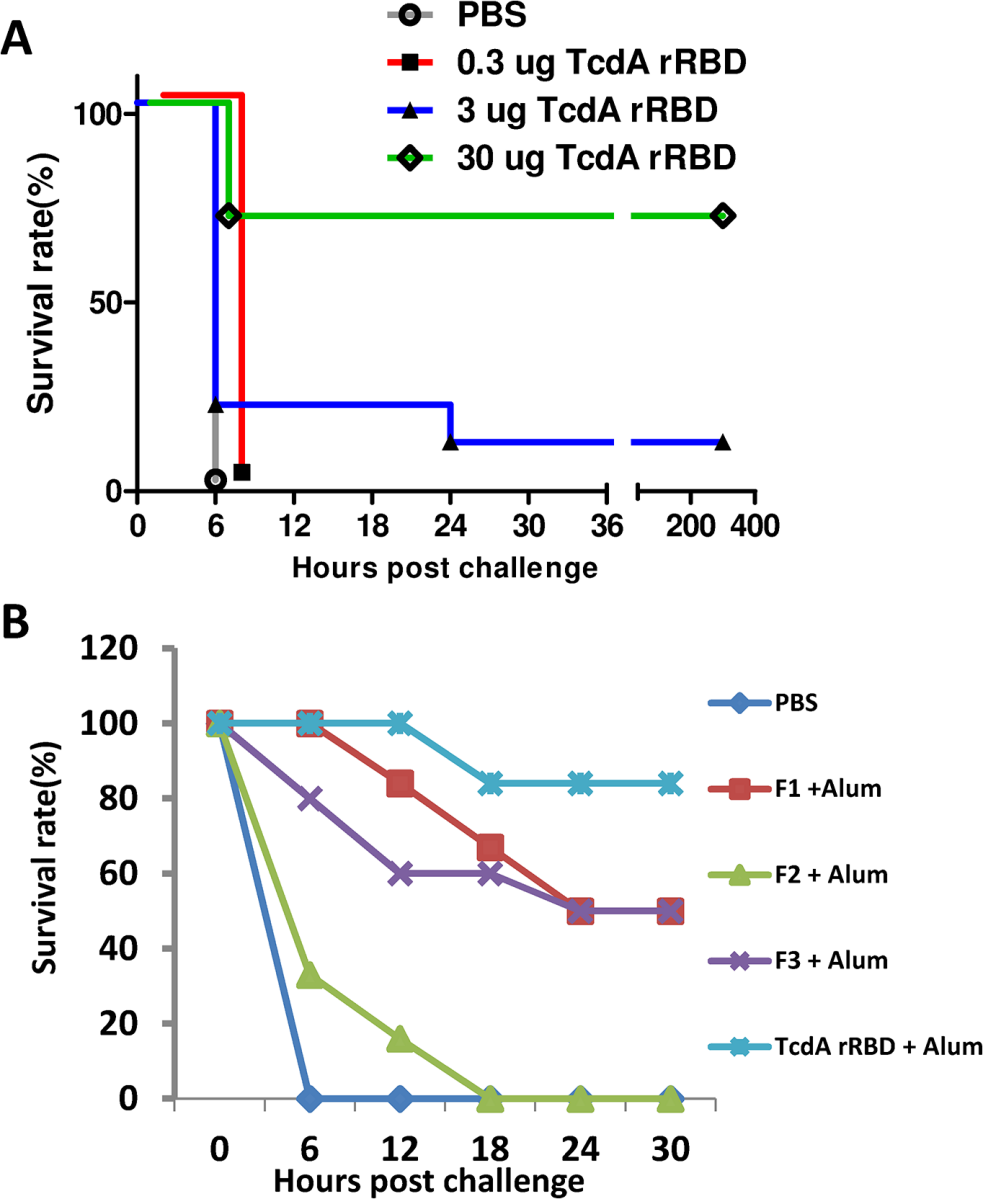
Protection of TcdA rRBD against lethal toxin A challenge. (A) BALB/c mice were challenged with a lethal dose of toxin A two weeks after three immunizations with TcdA rRBD (0.3, 3, and 30 μg) via intramuscular injection. (B) BALB/c mice were challenged with a lethal dose of toxin A two weeks after 3 × 10 μg of PBS, TcdA rRBD, or F1, F2 or F3 formulated with alum by intramuscular injection. The survival rate is reported at the defined time.

To further evaluate the role of this anti-toxin neutralizing activity *in vivo*, mice were immunized 3 times with increasing doses of TcdA rRBD (0.3, 3, or 30 μg) and challenged 5 times with the lethal dose 50 (LD_50_) of toxin A (Fig 9A). A low dose vaccination (0.3 μg) induced good anti-RBD antibody responses (titer = 3 × 10^3^) that were not protective (Fig 9A), although NT = 8 was identified in the pooled sera. The sera obtained from mice vaccinated with 3 and 30 μg of TcdA rRBD had NT = 32 and 128, respectively, but their protection rates were 17% and 67%, respectively (Fig 9A and Table 1). When mice vaccinated with 10 μg of F1, F2 or F3 formulated with alum were challenged with 5× LD_50_ of TcdA, the survival rates were 50%, 0%, and 50% for the F1, F2 and F3 groups, respectively (Fig 9B). This 50% protection rate may be attributable to strong neutralizing antibody responses (NT = 64) induced by F1 and F3 formulated in alum, whereas the neutralizing antibody responses induced by F2 formulated in alum remained low (NT = 16) and provided no protection (Table 1). Interestingly, mice immunized with 10 μg of TcdA rRBD formulated with alum demonstrated 83% protection against a TcdA challenge, and the neutralization titer was 128. Taken together, the present results suggest that protective immune responses are correlated with anti-toxin neutralization titers (Table 1). As TcdA rRBD, F1 and F3 formulated with alum can elicit strong immune protective responses against *C*. *difficile* toxin A, they should be considered as components in CDI subunit vaccines.

### Adjuvant properties of TcdA rRBD and its fragments

**Fig 10:**
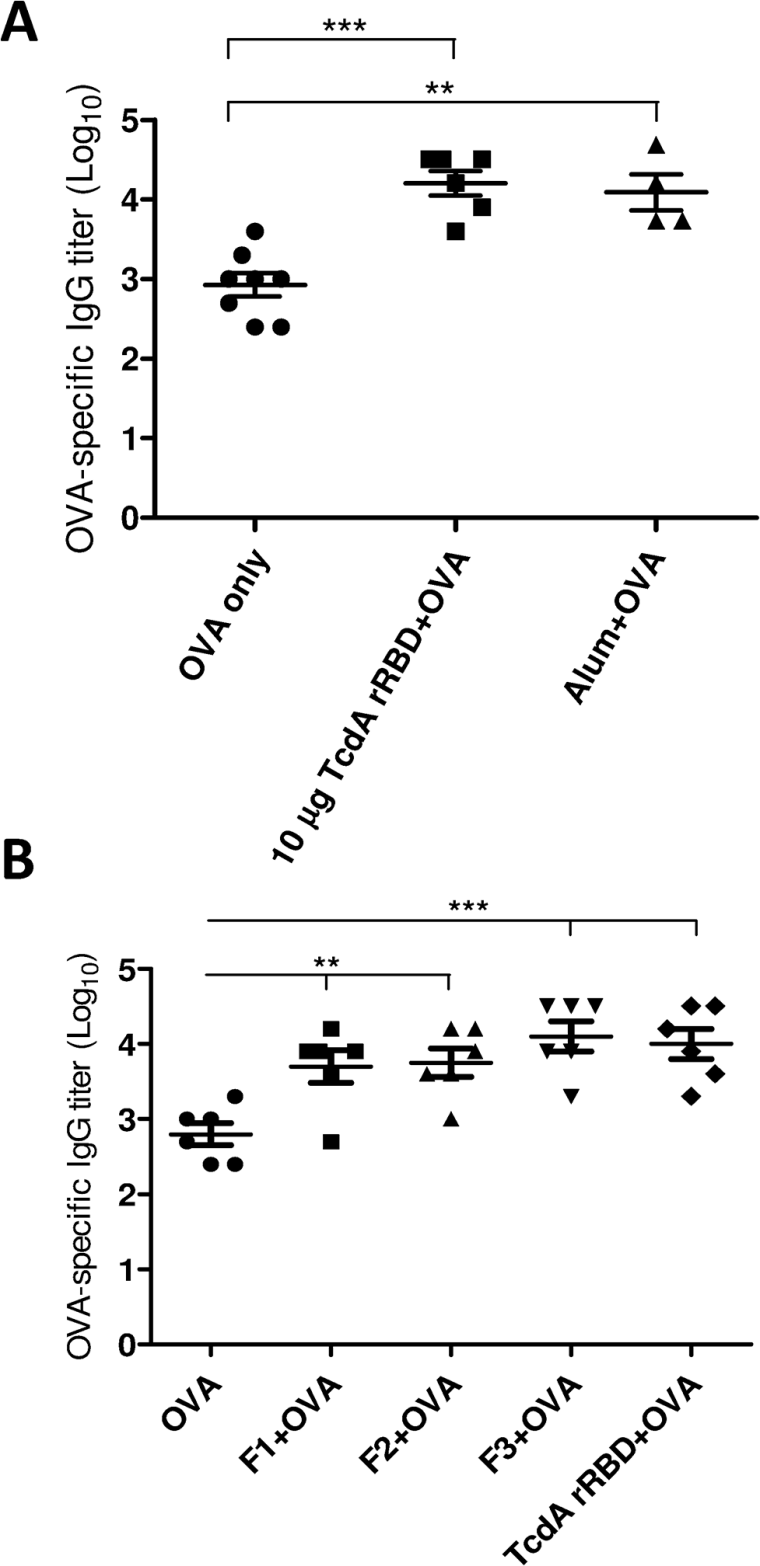
Adjuvant effects of TcdA rRBD and its truncated fragments. To demonstrate the adjuvant effects of TcdA rRBD, enhancement of the anti-OVA IgG response was evaluated by co-administration of TcdA rRBD and OVA. (A) BALB/c mice were immunized with 2 × 2 μg of OVA formulated with or without 10 μg of TcdA rRBD and alum as a positive control. (B) BALB/c mice were immunized with 2 μg of OVA formulated with 10 μg of TcdA rRBD, F1, F2, or F3. The anti-OVA IgG titer was determined by OVA-specific ELISA. The symbols ** and *** indicate *p*<0.01 and *p*<0.005, respectively.

Previous studies [43–44] have reported that the C-terminal part of TcdA could serve as a mucosal adjuvant to enhance the immune response toward a co-administered antigen, and the present study also demonstrated that TcdA rRBD is capable of promoting the maturation of dendritic cells and inducing strong functional antibody responses; these factors prompted us to investigate whether TcdA rRBD and its fragments could be used as an adjuvant. A ten-fold increase in anti-OVA IgG titers (~1 × 10^4^) was observed in the mouse group immunized with OVA (2 μg) formulated with TcdA rRBD (10 μg) compared to titers obtained with OVA alone (Fig 10A). The mouse immunogenicity studies were repeated with OVA formulated with either 0.3 or 3 μg of TcdA rRBD. Interestingly, even at a dose as low as 0.3 μg, TcdA rRBD induced at least a 10-fold increase in OVA-specific IgG titers over those obtained with OVA alone (S6 Fig). The increase in anti-OVA responses was statistically significant (*p*<0.05). There was no significant difference in the immunogenicity of OVA formulated in TcdA rRBD or alum (Fig 10A). Interestingly, antisera obtained from mice vaccinated 2 times with 2 μg of OVA formulated with 10 μg of F1, F2, or F3 also demonstrated at least a ten-fold increase in anti-OVA IgG titers over those obtained with OVA alone (*p*<0.01) (Fig 10B). In addition, there was an insignificant difference in adjuvant activity between F1, F2, F3 and TcdA rRBD (Fig 10A). These results indicate that TcdA rRBD and its fragments could be individually used as potent adjuvants for the intramuscular immunization route to enhance immune responses against weak immunogens.

## Discussion

To control the increasing incidence of hospital-acquired CDI and curb excessive medical costs, RBD-based CDI vaccines are being developed because recombinant subunit vaccines are cost-effective [11, 24–29, 45]. The clinical isolates of *C*. *difficile* are rapidly evolving, but sequence analyses of *C*. *difficile* strains deposited in the NCBI protein database reveal that the amino acid sequences of TcdA RBD share more than 97% identity, and new *C*. *difficile* strains are primarily BI/NAP1/027-related isolates [46]. To elicit broadly neutralizing antibodies against TcdA, a consensus sequence of TcdA RBD was derived from the NCBI protein database and was found to be identical to the *C*. *difficile* R20291 strain, a ribotype 027 strain. To study the specific roles and functions of the 7 putative receptor-binding regions, TcdA RBD was divided into three fragments: F1, F2 and F3. Each fragment was purposely designed to contain 3 potential lectin-like receptor-binding (LR) sites. F1 and F2 contain an overlapping LR site #3; F2 and F3 have an overlapping LR site #5 (Fig 1C). It should be noted that the consensus sequence and VPI 10463 reference strain used in all functional assays in this study have 33 synonymous changes within the RBD, including 11, 24 and 16 different amino acids in the F1, F2 and F3 fragments, respectively; these changes are highlighted in Fig 1A. In addition, 12 changes were found within the 7 potential lectin-binding sites, including 1, 5, 3, 2 and 1 amino acids changed in LRs #1, 3, 5, 6 and 7, respectively (Fig 1C). Most changes are found in F2, which may explain why F2 has less potent biochemical and immunological properties (Tables 1 and 2).

**Table 2:** Summary of biochemical properties of TcdA rRBD and RBD truncated fragments.

Antigen	Biological Properties				
	Cell binding (apparent K_d_ μM)#		HA activity(pmol)	Cellular uptake	DC maturation(μM)
	Vero cell	Caco-2 cell			
TcdA rRBD	0.14	0.09	0.4	++++	0.2
F1	0.26	0.18	No	+/-	0.8
F2	0.56	0.32	32	++	1
F3	0.16	0.08	2	++++	0.2
TcdA	ND	ND	2	Yes*	Yes*

# The apparent K_d_ is defined as the half-maximal binding of TcdA rRBD to the receptors.

*Yes indicates the information was derived from the literature [40–42, 48].

ND indicates not performed.

The biological and immunological properties of TcdA RBD and its fragments were evaluated using several functional assays [34–36,47–48], and each fragment demonstrated certain interesting properties. The receptor-binding activities obtained from both the Vero/Caco-2 cell-binding assays and FACS analysis (Fig 3A to 3D) provide direct and strong evidence that TcdA rRBD and its fragments (rRBD, F1, F2 and F3) are correctly folded (S1 Fig). These results are consistent with other reports showing that the fragments of RBD containing 5 to 15 short repetitive sequences could form stable folded β-solenoid secondary structures [31]. A novel finding in the present study is that F1, which contains the first three LR sites, may consist of a unique receptor-binding domain that (a) binds to Vero and Caco-2 cells but is not internalized and (b) does not agglutinate rabbit erythrocytes but still has the ability to activate DC maturation and induce anti-TcdA neutralizing antibody responses. The biochemical properties of F3, which are consistent with other reports [24, 29, 35, 43–45, 47–49], indicate that the last three LR sites contribute to strong HA activity in the rabbit red blood cell agglutination assay, fast and strong binding to both Vero and Caco-2 cells, as shown by FACS analysis, and potent adjuvant activity in enhancing anti-OVA IgG antibody responses. Its rapid internalization properties suggest that F3 could serve as a non-toxic carrier for new drug or vaccine delivery systems with important pharmaceutical applications. F2 possesses lower biological and immunological activities, indicating that the 8 amino acid changes in LR sites #3 (367V→G; 380H→D; 381N→A; 394K→E) and 5 (618D→N; 629H→D; 630N→A; 699Y→S) may be important for the biological and immunological properties of the RBD. Based on the results, these changes (i) affected and reduced the binding between F2 and the monoclonal antibody PCG-4 (Fig 2D), which recognizes an epitope located within the F2 fragment [50]; (ii) might have decreased F2 cell-binding activity (Fig 3D) and HA activity (Table 2); and (iii) certainly influenced the quality of the functional antibodies induced by F2 in the animal immunogenicity study in which F2 elicited no protection against TcdA (VPI10463 strain) challenge (Table 1).

The Vero and Caco-2 cell-binding assays with FACS indicated that TcdA rRBD and its fragments possess different binding activities, and as shown in Fig 2D, the TcdA-specific monoclonal antibody PCG-4 recognized F2 the least. To eliminate the antibody binding affinity problem, FACS analyses were repeated with an anti-His tag antibody, and similar results were obtained (data not shown). This result confirmed that the Vero and Caco-2 cell-binding activities are as follows: F3>F2>F1 (see the insert in Fig 3D).

The HA assay reflects the binding activities of each TcdA rRBD fragment to rabbit erythrocytes. Our current results further demonstrate that the N-terminal (F1) and middle (F2) regions of TcdA rRBD exhibit no or poor HA activity in rabbit erythrocytes. Because F1 has no HA activity and did not internalize after cell binding, F1 will be an excellent choice for the identification of specific cell receptor(s). In addition, we hypothesize that the putative carbohydrate-binding sites LR1/LR2 in F1 and LR6/LR7 in F3 bind to different ligands on the cell surface and may amplify receptor-binding affinity through a multivalent ligand mechanism. Therefore, F1 and F3 should be further truncated to investigate the specific roles and functions of the 7 putative receptor-binding sites of RBD.

TcdA, TcdA RBD and cholera toxin have been shown to possess mucosal adjuvant activity [42–44, 49, 51–53]. The present study has demonstrated that TcdA rRBD and its fragments can individually act as potent adjuvants in the intramuscular immunization route to enhance immune responses against OVA. We also clearly show that 2 μM of either TcdA rRBD or its truncated fragments is sufficient to promote the maturation of BMDCs and increase the secretion of pro-inflammatory cytokines (Figs 6 and 7). CT has been found to essentially promote DC maturation in the presence of its catalytic domain [49–50], but our current results clearly illustrate that TcdA rRBD and its fragments do not require the N-terminal catalytic domain and transmembrane domain of TcdA for DC activation. TcdA rRBD and its fragments have exhibited different levels of adjuvant activity in DC activation and the secretion of pro-inflammatory cytokines. F1 and F2 represent low DC activity, but they alone still possess strong adjuvant activity to elicit anti-RBD antibody responses and enhance immune responses to OVA through a T-cell bystander mechanism. Therefore, the DC activity of the TcdA rRBD fragments may not be the only route to modulate the immune system. Additionally, several reports have shown that TcdA can modulate various immune cells [51–55]. Further studies should be performed to determine whether TcdA rRBD is also involved in a more complex immune system network.

The present immunogenicity studies show that only toxoid A immunization induces the strongest TcdA neutralization titers and 100% protection (Table 1). The TcdA rRBD and/or rRBD fragments only induce partial protection in the TcdA mouse challenge model. These differences in protection levels can be explained by the following reasons. One is that other regions in TcdA are important and involved in protection against the toxin in the mouse challenge model and may be required for vaccine development. Another reason is that the amino acid sequences of immunogens (TcdA rRBD and its fragments) are derived from the 027 strain and are different from the challenge VPI 10463 strain. Thirty-three amino acid changes within RBD (Fig 1A) may be sufficient to influence the induction of a protective functional cross-neutralizing antibody response. In addition, a low dosage (10 μg) of TcdA rRBD and its fragments was used in the present study compared to other previous reports, in which different dosages, adjuvants, immunization routes and/or vectors were used and could result in different levels of protection in the toxin mouse challenge model [22–29]. The current literature [24,29,36] has indicated that toxin A or toxin A-derived fragments alone cannot induce a protective immune response against live *C*. *difficile* bacteria in the hamster challenge model. In fact, our preliminary results (Huang et al., unpublished results) indicated that TcdA RBD combined with TcdB RBD can induce efficient protection against *C*. *difficile* bacteria challenge in hamster models.

Overall, the current study has demonstrated that the biochemical properties of F3, which are consistent with other reports [24, 29, 42–44], indicate that the last three LR sites contribute to strong HA activity in the rabbit red blood cell agglutination assay, rapid and strong binding to both Vero and Caco-2 cells, as shown by FACS analysis, and potent adjuvant activity to enhance anti-OVA IgG antibody responses. F1, F2 and F3 have similar short repetitive amino acid sequences and putative oligosaccharide-binding sites (Fig 1B and 1C) but have dissimilar biochemical and immunological properties. Future studies should investigate which amino acids in these repetitive sequences are responsible for their biochemical functions and TLR agonist activity. As TcdA rRBD, F1 and F3 can elicit immune protective responses against *C*. *difficile* toxin A, TcdA rRBD and its fragments are certainly potential components for future candidate vaccines against *C*. *difficile*-associated diseases.

## Supporting Information

S1 Fig The secondary structure of TcdA rRBD and its fragments. (A) CD spectra of TcdA rRBD and its fragments (F1, F2 and F3). (B) The thermal stability curves of TcdA rRBD and its fragments analyzed using CD spectra based on the values at θ_230 nm_.
(PDF)

S2 Fig Vero cell-binding abilities of TcdA rRBD and its truncated fragments. After TcdA rRBD fragments were incubated with Vero cells for 30 min, the TcdA rRBD fragments were characterized by immunoblot analysis using an anti-TcdA specific monoclonal antibody. Two protein concentrations (2 and 6 μM) were used in the cell-binding assay.
(PDF)

S3 Fig Cellular uptake of TcdA rRBD, F1 and F3 through the clathrin-dependent pathway. Chlorpromazine (CPZ) was used to inhibit TcdA rRBD, F1 and F3 internalization. To confirm that the cellular uptake was receptor-mediated clathrin-dependent endocytosis, CPZ was added into the cell-binding medium, and TcdA rRBD, F1 and F3 internalization was inhibited. The internalization signals for TcdA rRBD and its fragments into Vero cells were evaluated by confocal microscopy at 5, 15, and 30 min. The images were collected from a single stack in the central region of the *z* axis. Green fluorescence signals represent the locations of TcdA rRBD, F1 and F3. Nuclei were stained with DAPI and are shown in blue.
(PDF)

S4 Fig Mouse anti-RBD antibody responses elicited by different dosages of TcdA rRBD. (A) BALB/c mice were immunized three times with 3, 10 or 30 μg of TcdA rRBD, and alum formulation served as the positive control. Anti-RBD titers at 0, 2, 4, 6, and 8 weeks were determined by RBD-specific ELISA. (B) Specific anti-RBD IgG isotypes and IgA were analyzed with the sera obtained from the 6th week post-immunization.
(PDF)

S5 Fig Immunogenicity study with TcdA rRBD and its truncated fragments with alum. BALB/c mouse anti-RBD antibody responses elicited by 3 × 10 μg of either TcdA rRBD or its fragments formulated with alum. Anti-RBD IgG titers at 6 weeks were determined by RBD-specific ELISA.
(PDF)

S6 Fig Adjuvant effects of TcdA rRBD. To demonstrate the adjuvant effects of TcdA-RBD, the increase in the anti-OVA IgG response was evaluated via co-administration of TcdA rRBD and OVA. BALB/c mice were immunized with 2 μg of OVA formulated with either 0.3 or 3 μg of TcdA rRBD or alum as a positive control. The anti-OVA IgG titer was determined by OVA-ELISA.
(PDF)

S1 File Supporting Information files for Figs 3, 4, 6, 7, 8, 9 & 10 are in the Compressed/ZIP File Archive. (ZIP)
