# Supplementary figures and images for: Biochemical and Immunological Characterization of Truncated Fragments of the Receptor-Binding Domains of C. difficile Toxin A
Source: PLoS One. 2015 Aug 13;10(8):e0135045. doi: 10.1371/journal.pone.0135045 (PMC4536038; doi:10.1371/journal.pone.0135045)

S1 Fig.

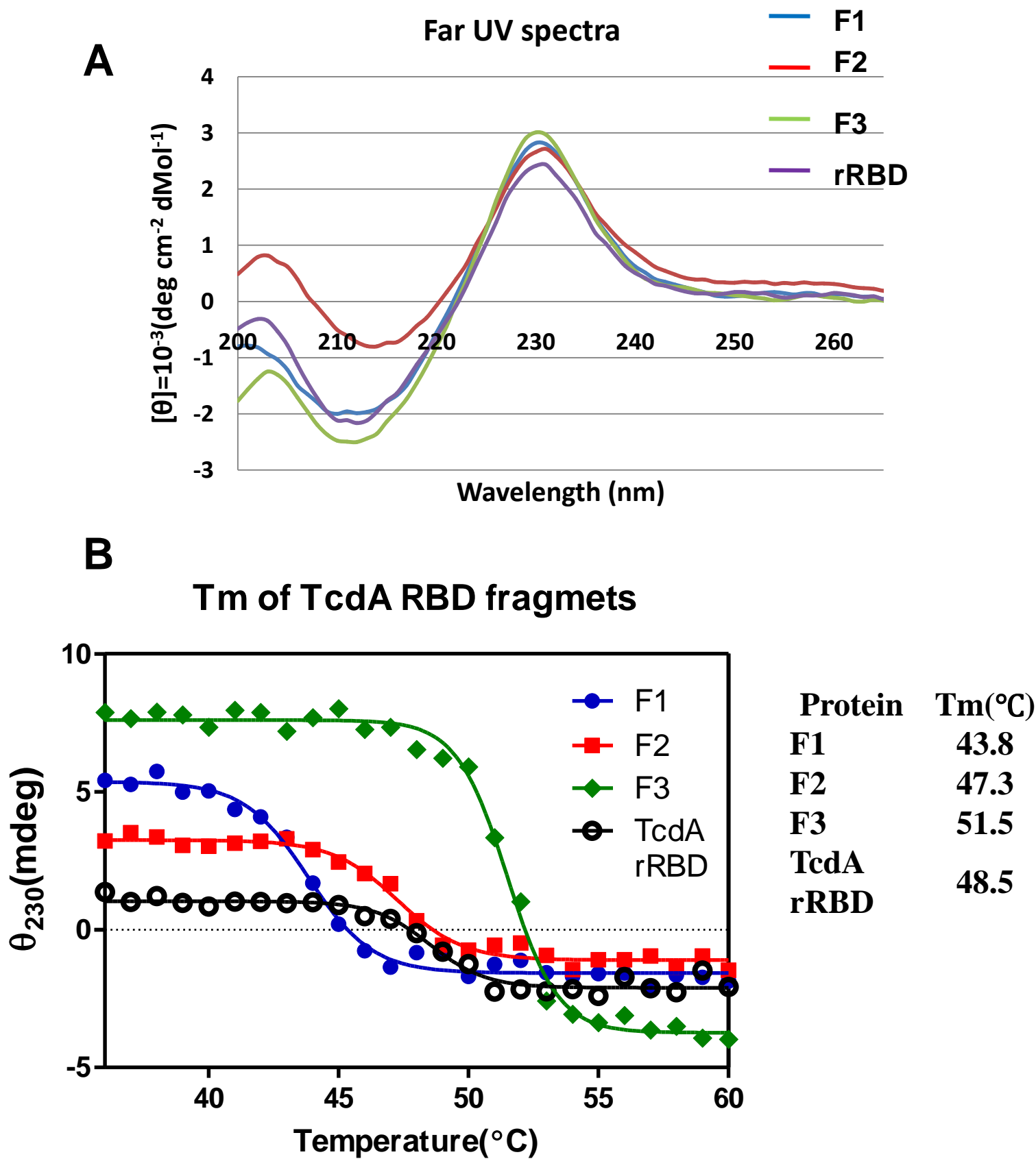

Supplement: S1 Fig — (A) CD spectra of TcdA rRBD and its fragments (F1, F2 and F3). (B) The thermal stability curves of TcdA rRBD and its fragments analyzed using CD spectra based on the values at θ230 nm. (PDF) [file pone.0135045.s001.pdf]

**S2 Fig.**

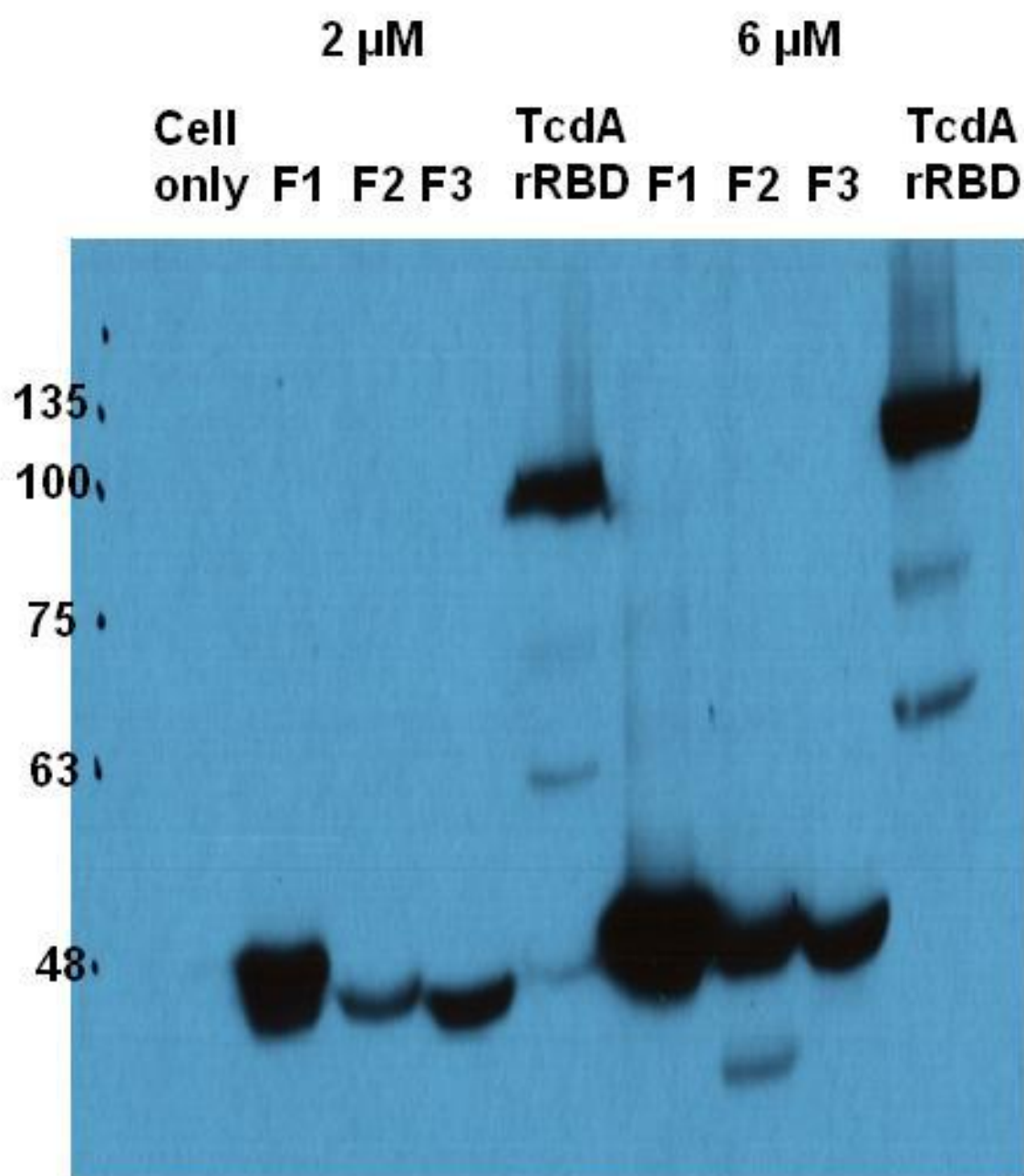

Supplement: S2 Fig — After TcdA rRBD fragments were incubated with Vero cells for 30 min, the TcdA rRBD fragments were characterized by immunoblot analysis using an anti-TcdA specific monoclonal antibody. Two protein concentrations (2 and 6 μM) were used in the cell-binding assay. (PDF) [file pone.0135045.s002.pdf]

**S3 Fig.**

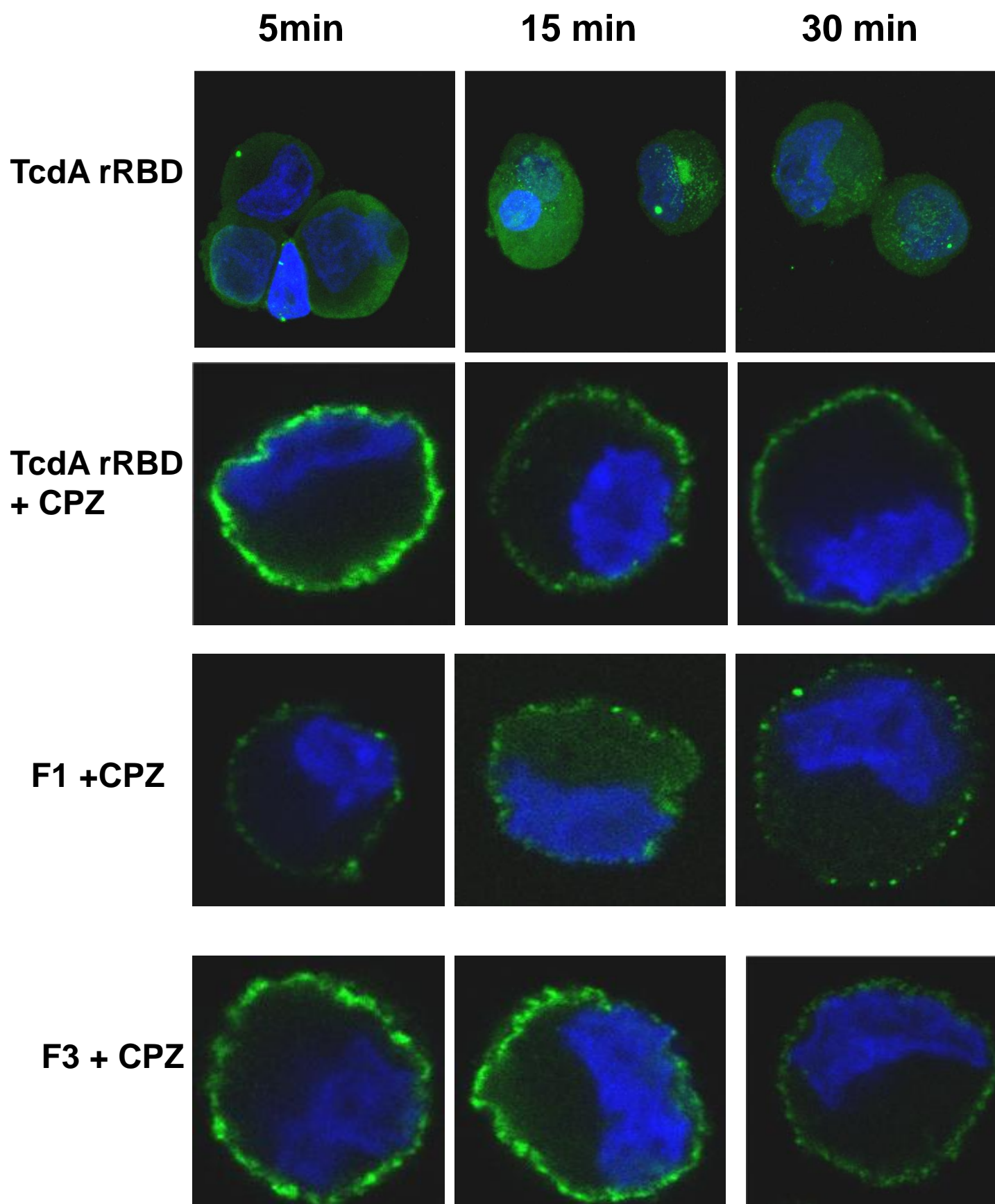

Supplement: S3 Fig — Chlorpromazine (CPZ) was used to inhibit TcdA rRBD, F1 and F3 internalization. To confirm that the cellular uptake was receptor-mediated clathrin-dependent endocytosis, CPZ was added into the cell-binding medium, and TcdA rRBD, F1 and F3 internalization was inhibited. The internalization signals for TcdA rRBD and its fragments into Vero cells were evaluated by confocal microscopy at 5, 15, and 30 min. The images were collected from a single stack in the central region of the z axis. Green fluorescence signals represent the locations of TcdA rRBD, F1 and F3. Nuclei were stained with DAPI and are shown in blue. (PDF) [file pone.0135045.s003.pdf]

S4 Fig.

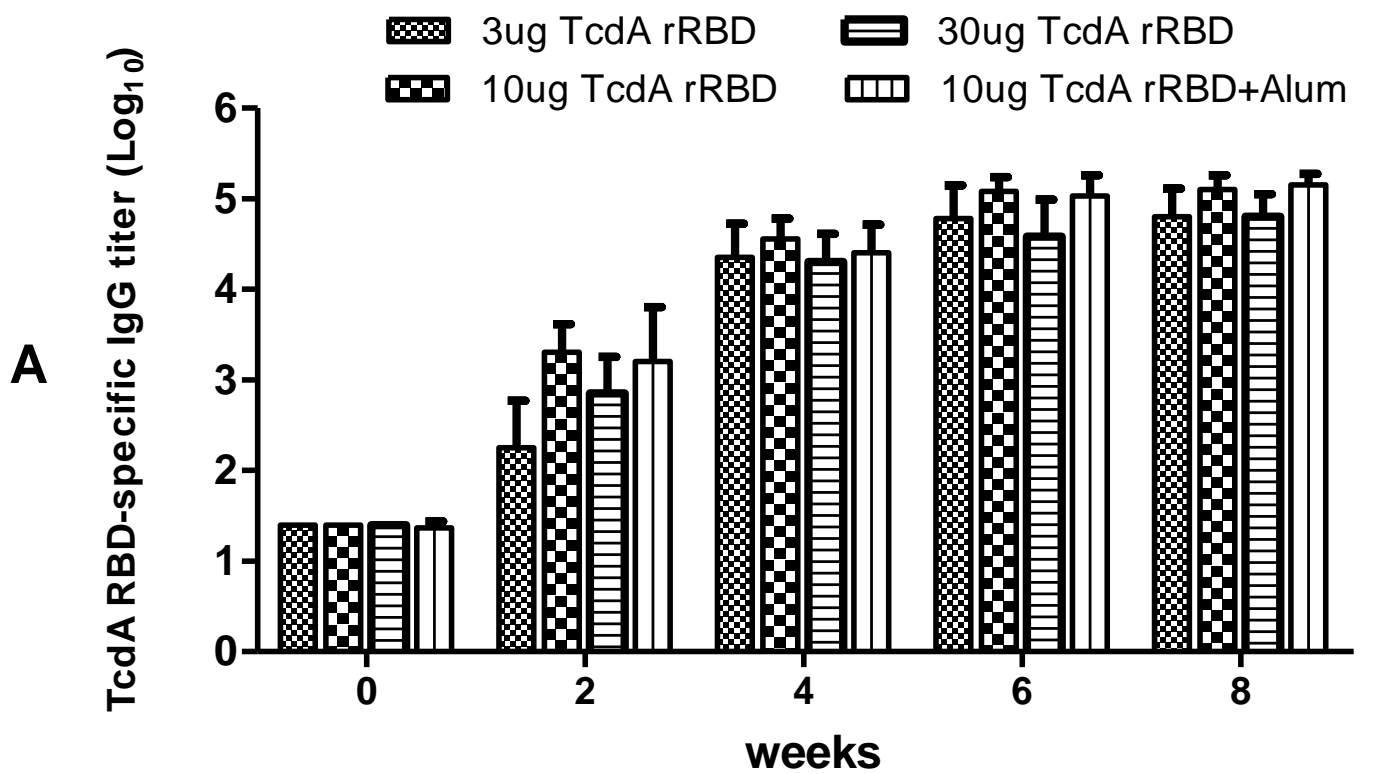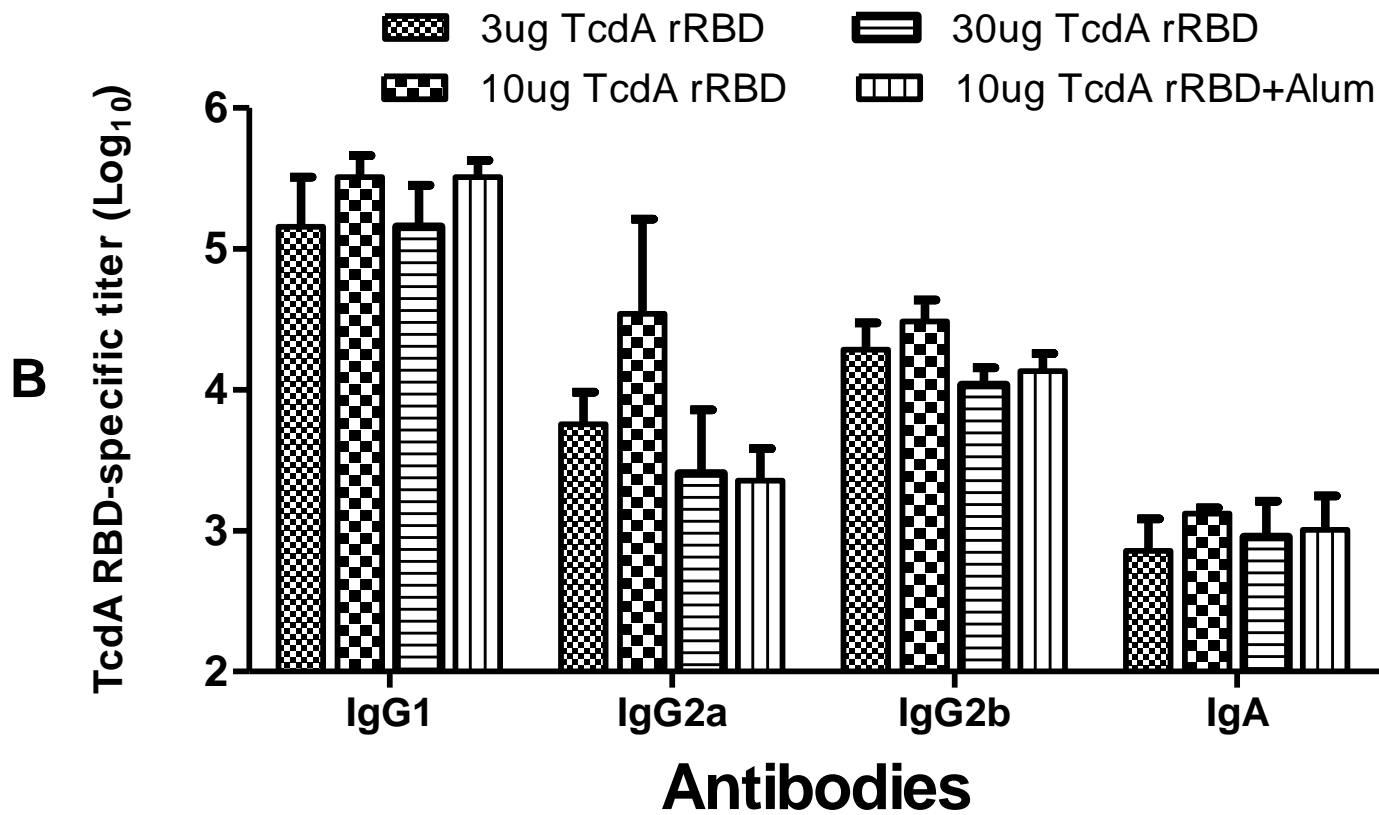

Supplement: S4 Fig — (A) BALB/c mice were immunized three times with 3, 10 or 30 μg of TcdA rRBD, and alum formulation served as the positive control. Anti-RBD titers at 0, 2, 4, 6, and 8 weeks were determined by RBD-specific ELISA. (B) Specific anti-RBD IgG isotypes and IgA were analyzed with the sera obtained from the 6th week post-immunization. (PDF) [file pone.0135045.s004.pdf]

S5 Fig.

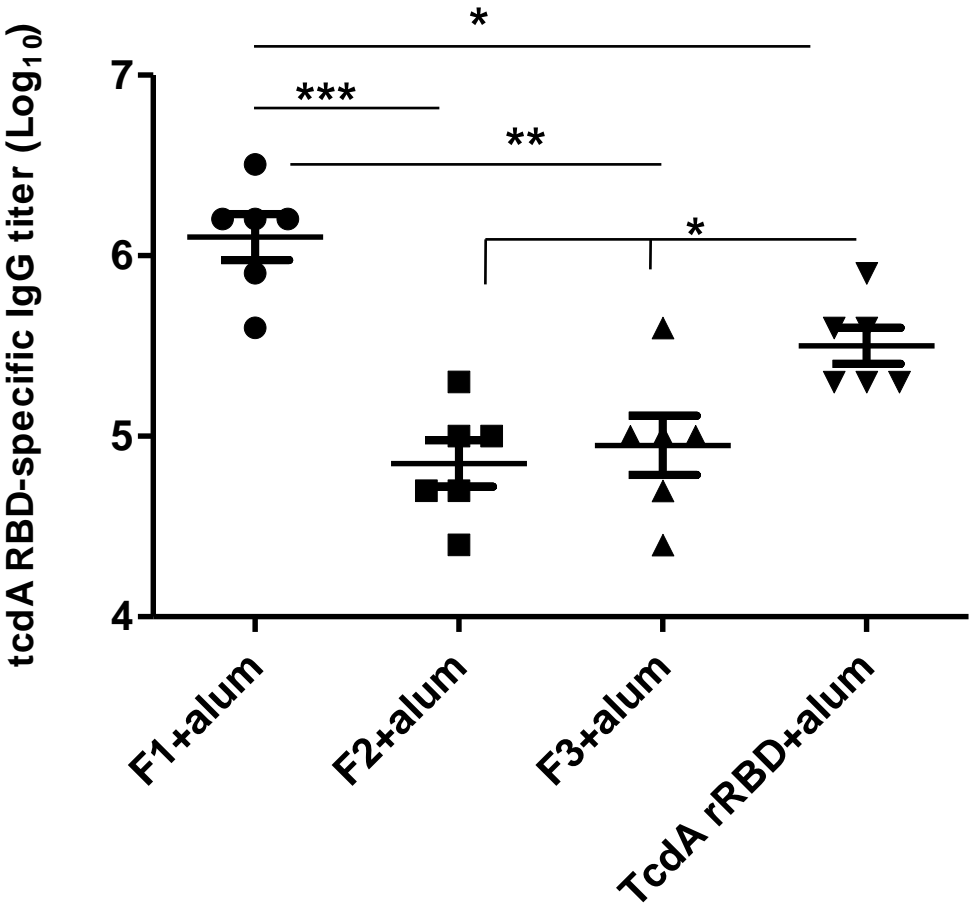

Supplement: S5 Fig — BALB/c mouse anti-RBD antibody responses elicited by 3 × 10 μg of either TcdA rRBD or its fragments formulated with alum. Anti-RBD IgG titers at 6 weeks were determined by RBD-specific ELISA. (PDF) [file pone.0135045.s005.pdf]

S6 Fig.

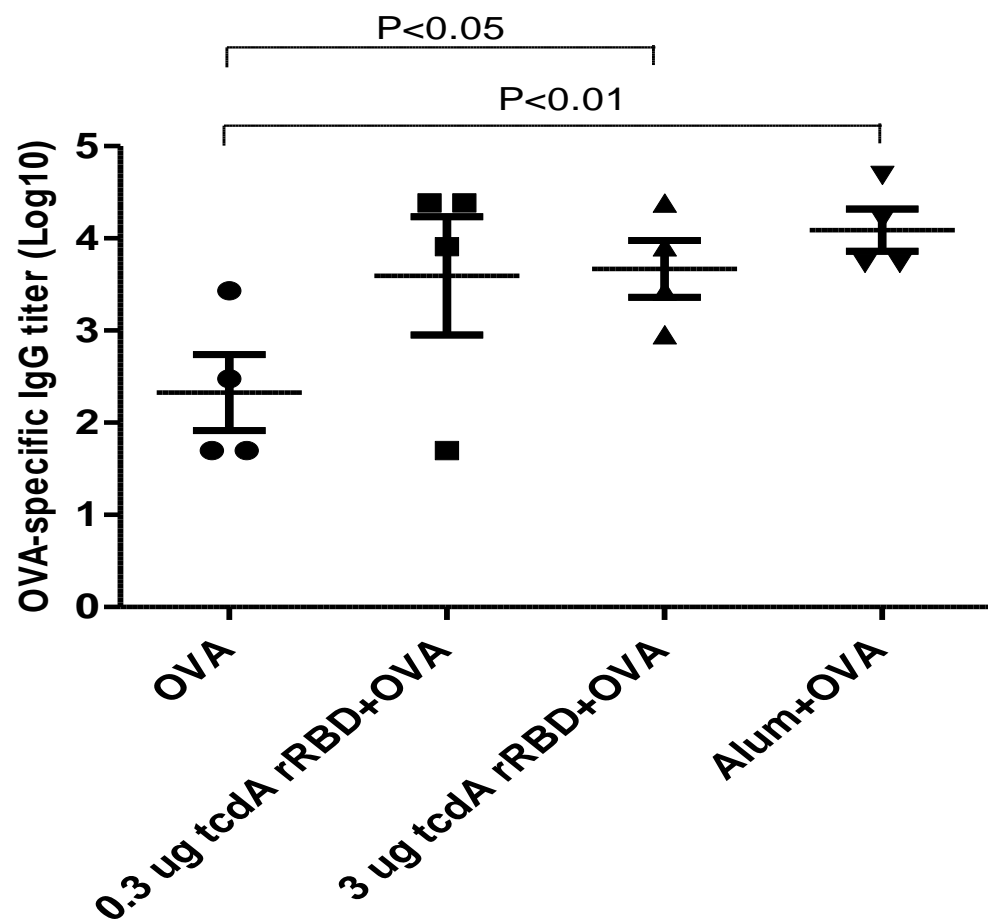

Supplement: S6 Fig — To demonstrate the adjuvant effects of TcdA-RBD, the increase in the anti-OVA IgG response was evaluated via co-administration of TcdA rRBD and OVA. BALB/c mice were immunized with 2 μg of OVA formulated with either 0.3 or 3 μg of TcdA rRBD or alum as a positive control. The anti-OVA IgG titer was determined by OVA-ELISA. (PDF) [file pone.0135045.s006.pdf]
